# Lymphedema alters lipolytic, lipogenic, immune and angiogenic properties of adipose tissue: a hypothesis-generating study in breast cancer survivors

**DOI:** 10.1038/s41598-021-87494-3

**Published:** 2021-04-14

**Authors:** Michal Koc, Martin Wald, Zuzana Varaliová, Barbora Ondrůjová, Terezie Čížková, Milan Brychta, Jana Kračmerová, Lenka Beranová, Jan Pala, Veronika Šrámková, Michaela Šiklová, Jan Gojda, Lenka Rossmeislová

**Affiliations:** 1 Department of Pathophysiology, Centre for Research On Nutrition, Metabolism and Diabetes, Third Faculty of Medicine, Charles University, Ruská 87, 100 00 Prague 10, Czech Republic; 2 Department of Surgery, Second Faculty of Medicine, Charles University and Motol University Hospital, Prague 5, Czech Republic; 3 Department of Radiotherapy and Oncology, Kralovske Vinohrady University Hospital, Prague 10, Czech Republic; 4 Franco-Czech Laboratory for Clinical Research on Obesity, Third Faculty of Medicine, Prague 10, Czech Republic; 5 Second Internal Medicine Department, Kralovske Vinohrady University Hospital, Prague 10, Czech Republic

**Keywords:** Cardiovascular biology, Cell signalling, Cancer, Cardiovascular diseases, Mesenchymal stem cells, Reprogramming, Stem-cell differentiation, Cytokines, Inflammation, Lymphatic system, Lymphocytes, Cell division, Non-coding RNAs, Transcription, Angiogenesis, Cardiovascular biology, Metabolism, Fat metabolism, Cell biology, Immunology, Molecular biology, Physiology, Stem cells, Cardiology, Diseases

## Abstract

Later stages of secondary lymphedema are associated with the massive deposition of adipose tissue (AT). The factors driving lymphedema-associated AT (LAT) expansion in humans remain rather elusive. We hypothesized that LAT expansion could be based on alterations of metabolic, adipogenic, immune and/or angiogenic qualities of AT. AT samples were acquired from upper limbs of 11 women with unilateral breast cancer-related lymphedema and 11 healthy women without lymphedema. Additional control group of 11 female breast cancer survivors without lymphedema was used to assess systemic effects of lymphedema. AT was analysed for adipocyte size, lipolysis, angiogenesis, secretion of cytokines, immune and stem cell content and mRNA gene expression. Further, adipose precursors were isolated and tested for their proliferative and adipogenic capacity. The effect of undrained LAT- derived fluid on adipogenesis was also examined. Lymphedema did not have apparent systemic effect on metabolism and cytokine levels, but it was linked with higher lymphocyte numbers and altered levels of several miRNAs in blood. LAT showed higher basal lipolysis, (lymph)angiogenic capacity and secretion of inflammatory cytokines when compared to healthy AT. LAT contained more activated CD4+ T lymphocytes than healthy AT. mRNA levels of (lymph)angiogenic markers were deregulated in LAT and correlated with markers of lipolysis. In vitro, adipose cells derived from LAT did not differ in their proliferative, adipogenic, lipogenic and lipolytic potential from cells derived from healthy AT. Nevertheless, exposition of preadipocytes to LAT-derived fluid improved their adipogenic conversion when compared with the effect of serum. This study presents results of first complex analysis of LAT from upper limb of breast cancer survivors. Identified LAT alterations indicate a possible link between (lymph)angiogenesis and lipolysis. In addition, our in vitro results imply that AT expansion in lymphedema could be driven partially by exposition of adipose precursors to undrained LAT-derived fluid.

## Introduction

Breast cancer related secondary lymphedema is a chronic disorder based on lymphatic insufficiency that may develop after anticancer therapy consisting of lymph node dissection, chemotherapy or irradiation^1^. Damage of the lymphatic system then leads to interstitial fluid accumulation, lymph leakage and backflow in the affected area. Lymphedema is in later stages associated with the massive deposition of adipose tissue (AT)^2,3^. Although the factors driving this lymphedema-associated AT (LAT) expansion in humans remain still rather elusive, the causal link between defects in lymphatic system and aberrant AT accumulation was demonstrated in several animal models. Chy mice, an animal model of human Milroy’s disease (primary lymphedema), have enhanced AT accumulation in subcutaneous layer^4^. Also mice with heterozygous inactivation of Prox-1, a master regulator of lymphatic vasculature, develop obesity and abnormal AT accumulation around the leaky mesenteric lymphatic vessels^5^. Similarly, abnormal accumulation of mesenteric AT in the Crohn’s disease -so called creeping fat- can be the consequence of profound defects in lymphatic drainage of the inflamed intestines^6^. Moreover, murine/rabbit mesenteric chylous lymph directly stimulates adipogenesis in vitro in corresponding preadipocytes^5,7^. Besides, murine stem cells from LAT exert higher expression of adipogenic markers (C/EBP, PPARγ) compared to the healthy AT^8,9^ and a pilot study on limited number of human samples showed that stem cells isolated from LAT have higher adipogenic capacity over stem cells from healthy individuals^10^. Importantly, acute damage to lymphatics in mice leads also to aberrant activation of immune cells—in fact, lymphedema-associated AT accumulation appears to be enhanced by CD4+ T cells that secrete mediators promoting growth of dysfunctional and thus leaky lymphatic vessels^11,12^. Together, several lines of evidence suggest that the leaking lymph or undrained interstitial fluid modify microenvironment and consequently various qualities of AT thus eventually promoting AT expansion. However, this concept has been studied mostly in experimental animals and thus the validity of this concept in humans still needs to be tested.

Therefore, the main aim of this study was to evaluate whether AT qualities possibly contributing to LAT accumulation are affected by breast cancer related secondary lymphedema in human. Thus we compared adipogenic, angiogenic, immune and metabolic properties of healthy and lymphedema-associated AT and adipose precursors derived from these tissues.

## Results

### Subjects characteristics

3 groups of women matched for weight were recruited: (i) 11 women with unilateral lymphedema in upper extremity (non-pitting breast cancer related lymphedema)-LYM group; (ii) 11 healthy women without lymphedema undergoing elective liposuction-Healthy group; (iii) 11 women- breast cancer survivors without lymphedema-NOLYM group. Women from LYM group developed lymphedema 0–13 years after primary anti-tumour treatment (median 2.5 years) and lymphedema lasted for 2–9 years (median 4.5 years) until they were indicated for liposuction. None of the women had active cancer. The amount of fat removed by liposuction was 869–2323 ml (median 1181 ml) and it represented 26–103% reduction of diseased limb volume compared to the volume of healthy limb (median 68%). Despite all the effort to achieve initial aim of matching, healthy women undergoing elective liposuction were younger and less obese than cancer survivors (both LYM and NOLYM groups) (Table 1). Healthy and LYM groups were matched according to relative fat mass measured by bioimpedance, but discordant in calculated relative fat mass (Table 1). This discrepancy apparently reflects higher volume of extracellular water in lymphedema patients, which has a direct impact on bioimpedance measurements. Healthy women had similar profile of blood lipids as LYM and NOLYM group but better indices of glucose metabolism (fasting glucose, insulin levels, HOMA-IR, Table 1), which could be related to the age and adiposity.

**Table 1:** Anthropometric and biochemical characteristics of the subjects. Data are shown as mean ± SD. *p < 0.05, **p < 0.01, ***p < 0.001 (difference related to healthy subjects).

	Healthy subjects (n = 11)	Subjects with secondary lymphedema (n = 11)	Cancer survivors without lymphedema (n = 11)
Age (year)	45.0 ± 8.1	60.4 ± 10.3***	66.1 ± 5.9 ***
Weight (kg)	69.2 ± 8.3	78.8 ± 13.1	78.3 ± 7.6
BMI (kg/m^2^)	25.2 ± 3.7	29.4 ± 4.0*	29.4 ± 3.5*
Fat mass (%)	32.5 ± 7.8	36.9 ± 5.3	41.1 ± 3.3**
Fat mass (%) calculated	32.1 ± 6.1	40.4 ± 5.4**	41.2 ± 5.4**
Fat mass (kg)	22.6 ± 7.1	28.7 ± 6.2	32.4 ± 5.9**
Fat mass (kg) calculated	22.5 ± 6.2	32.4 ± 9.2*	32.4 ± 6.6*
Waist circumference (cm)	83.6 ± 9.8	100.2 ± 10.4**	94.0 ± 11.0
Hip circumference (cm)	104.1 ± 6.8	112.1 ± 8.9	106.6 ± 7.6
Waist to Hip ratio	0.80 ± 0.07	0.89 ± 0.05*	0.88 ± 0.06*
FFA (mmol/l)	0.81 ± 0.29	0.80 ± 0.31	0.87 ± 0.29
Glycerol (µmol/l)	55.0 ± 31.9	78.8 ± 42.9	81.0 ± 43.2
Cholesterol (mmol/l)	4.90 ± 0.73	4.95 ± 0.72	5.27 ± 0.74
HDL cholesterol (mmol/l)	1.84 ± 0.41	1.50 ± 0.39	1.45 ± 0.27*
LDL cholesterol (mmol/l)	2.66 ± 0.78	2.81 ± 0.172	3.33 ± 0.65
Triglycerides (mmol/l)	0.88 ± 0.38	1.60 ± 0.56	1.79 ± 1.03*
Fasting glucose (mmol/l)	4.8 ± 0.4	5.9 ± 1.0**	5.9 ± 0.7**
Fasting insulin (mU/l)	4.3 ± 3.2	12.8 ± 6.2**	13.0 ± 5.3**
HOMA-IR	0.85 ± 0.79	3.53 ± 2.40**	3.52 ± 1.72***
Uric acid μmol/l	252.6 ± 59.9	339.1 ± 39.2*	295.7 ± 103.9

### Systemic effects of lymphedema

To assess possible systemic changes induced by lymphedema, we analysed relative content of immune cells in blood and plasma/serum levels of various cytokines and miRNAs.

**Figure 1:**
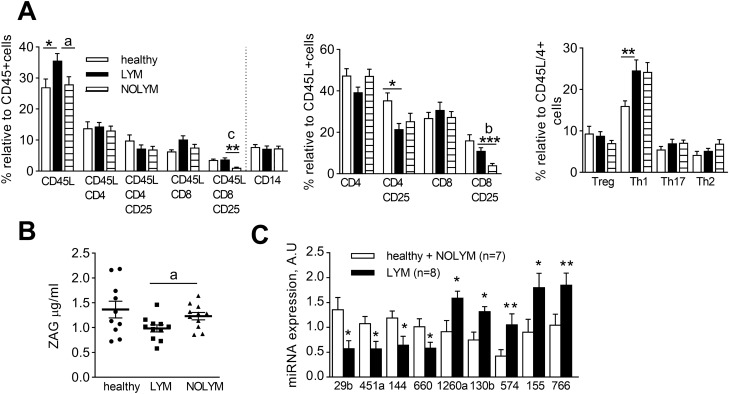
Systemic effects of lymphedema. (**A**) Relative content of immune cell populations in peripheral blood, expressed as percentage of CD45+ positive cells, CD45+ positive cells within lymphogate or CD45/CD4+ positive cells within lymphogate (ANOVA of LN transformed data, Bonferroni correction for post-hoc analysis, comparison to LYM group, *p < 0.05, **p < 0.01, Mann Whitney test comparison LYM vs. NOLYM, a- p < 0.05, b- p < 0.01, c- p < 0.001). (**B**) Concentration of ZAG in plasma (Mann–Whitney test, comparison LYM vs. NOLYM, a-p < 0.05). (**C**) Levels of selected miRNA in serum. Data are presented as expression levels relative to maximal expression for each miRNA (non-parametric T-test of Log2 transformed data, *p < 0.05, **p < 0.01).

We found higher numbers of total lymphocytes in LYM compared to Healthy and NOLYM (difference from NOLYM reached p = 0.09) (Fig. 1A). Within lymphocytes, activated CD4/CD25+ T lymphocytes were less represented in LYM group compared to healthy women, while activated CD8/CD25+ T lymphocytes were fewer in NOLYM compared to LYM group. Nevertheless, relative content of these lymphocyte subpopulations did not distinguish LYM women from both control groups concurrently. The relative distribution of monocyte populations was similar in all groups (Fig. 1A and not shown).

In plasma we have measured levels of 14 cytokines representing markers of inflammation (IL6, IL8, TNFα), adiposity (leptin, adiponectin, FABP4), lipolysis (ZAG, PEDF), growth (EGF, FGF acidic), angiogenesis (EGF, angiopoietin 2, VEGFA, VEGFC) and mitochondrial stress (GDF15). Only levels of ZAG were lower in plasma from LYM group compared to NOLYM (Fig. 1B). FGF acidic and VEGFA were not detected in plasma.

Analysis of 175 species of miRNA from serum LYM patients vs. healthy combined with NOLYM subjects led to a detection of several miRNAs that were deregulated in lymphedema (Fig. 1 C). According to principal component analysis (PCA), expression levels of four from these miRNA—miR-1260a, miR-29b-3p, miR-130b-3p, miR-660-5p- were able to separate subjects from LYM and combined NOLYM/healthy group (Supplementary Fig. 1A).

### Adipocyte-related characteristics of AT from lymphedema and healthy subjects

In order to comprehensively evaluate AT alterations induced by iatrogenic damage to lymphatic system, we analysed several in vivo or ex vivo parameters of LAT reflecting and related to adipocyte qualities- adipocyte size, adipogenic, lipogenic, and lipolytic signature—and compared it to properties of healthy AT from healthy women and/or AT from healthy limb of LYM women.

Previous study evaluating histological sections of LAT showed hypertrophy of lymphedema adipocytes^13^. Nevertheless, our analysis of isolated adipocytes did not show any significant difference in the mean diameter of adipocytes in between healthy and LYM groups (88.8 ± 5.6 lymphedema, vs. 97.4 ± 3.3 um healthy; AT tissue was not acquired from NOLYM group from ethical reasons, see Methods)*.* Even more, comparison of frequency distribution of adipocytes revealed higher proportion of larger adipocytes (100–120 µm) in healthy AT compared to LAT (Supplementary Fig. 1B). Thus, results of size analysis of isolated adipocytes did not confirm hypertrophic nature of adipocytes within LAT.

**Figure 2:**
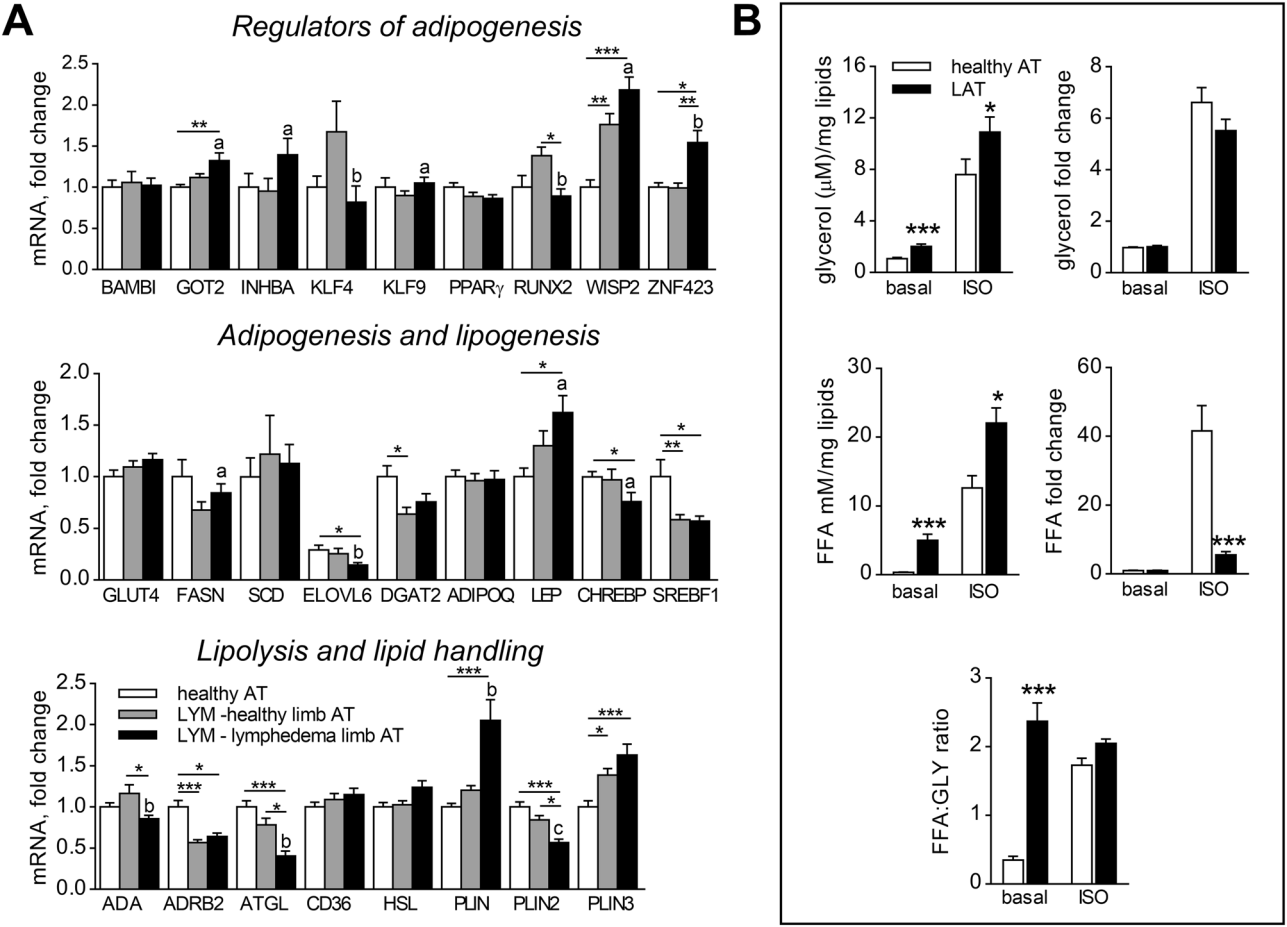
LAT qualities related to adipocytes. (**A**) mRNA levels of markers of adipogenesis, lipogenesis and lipolysis in whole AT expressed as fold change over the mean expression of healthy group (Kruskal–Wallis test of 2^ΔCt^ values, Dunn’s correction, comparison of 3 groups, *p < 0.05, **p < 0.01, ***p < 0.001; Wilcoxon test of paired healthy and diseased limb of LYM subjects, a- p < 0.05, b- p < 0.01, c- p < 0.001). (**B**) Ex vivo lipolysis in AT explants. Cells were exposed to basal conditions or 1 µM isoproterenol (ISO) for 4 h. Concentrations of glycerol and FFA normalized to mg of lipids and fold change over the basal conditions are shown (Two-way ANOVA of LN transformed data, Sidak post-hoc analysis, *p < 0.05, ***p < 0.001).

As previously documented, experimentally induced lymphedema in mice was connected with increased protein levels of proadipogenic transcription factors PPARγ and C/EBPα^8^. Therefore, we have evaluated mRNA expression of 9 factors implicated in human AT mass regulation and 7 genes involved in AT lipogenesis. This analysis revealed 5 genes that were altered in LAT compared to AT from both healthy subjects and healthy limb of LYM subject (Fig. 2A). Expression of adipogenic regulators revealed mixed adipogenic response to lymphedema, as mRNA expression of both proadipogenic (ZNF423) and anti-adipogenic (WISP2, GOT2) factors was higher in AT from diseased limb of LYM women when compared to AT from paired healthy limb and also to AT from healthy women. Moreover, expression of INHBA and KLF9 was enhanced while KLF4 and RUNX2 was decreased in paired comparison between healthy and diseased limb of LYM patients. Nevertheless, expression of adiposity marker- leptin- was increased in lymphedema limb compared to both healthy subjects and healthy limb of LYM subject. Similarly, this mixed response was observed also in the set of lipogenic markers- the expression of two analysed lipogenic markers (ChREBP, ELOVL6) was significantly lower in AT from diseased limb of LYM women when compared to AT from paired healthy limb and to AT from healthy women, while expression of FASN was higher in paired comparison between healthy and diseased limb of LYM patients (Fig. 2A).

Since altered lipolysis could contribute to AT expansion in lymphedema, we analysed basal and isoproterenol induced lipolysis rate ex vivo in AT explants and isolated adipocytes. Explants from lymphedema AT released more glycerol and FFA in both basal and isoproterenol stimulated conditions (Fig. 2B). The same trend for higher basal and isoproterenol stimulated glycerol levels in LYM compared to healthy was observed in isolated adipocytes from a subset of subjects (n = 6 healthy, n = 7 LYM) (Supplementary Fig. 1C). Although isoproterenol-induced lipolysis expressed as a fold change of basal lipolysis did not differ between the groups for glycerol, fold change of FFA was lower in LYM group compared to healthy. Moreover, baseline FFA:glycerol ratio in explants (but not isolated adipocytes) from LAT was much higher compared to healthy AT (Fig. 2B and Supplementary Fig. 1C), suggestive of lower re-esterification or utilization of FFA by LAT (adipocytes or SVF cells) within relatively intact AT structure.

We also analysed whether higher basal lipolysis in LAT is based on the alteration of expression of genes involved in lipolysis and lipid handling. Nevertheless, contrary to the expectations stemming from ex vivo results, ATGL mRNA expression was lower while expression of lipid droplet coating PLIN1 was higher in diseased limb compared to healthy and healthy limb of LYM (Fig. 2A).

### Immune characteristics of AT and related fibrosis and angiogenesis

Inflammation is a critical component in the pathophysiology of lymphedema^14^ and thus, it is feasible that lymphedema-associated AT deposition and alteration of AT metabolic function could be linked with higher inflammation. Therefore, we analysed immune cell content and related fibrotic and angiogenic properties-and compared it to properties of healthy AT from healthy women and/or AT from healthy limb of LYM women.

**Figure 3:**
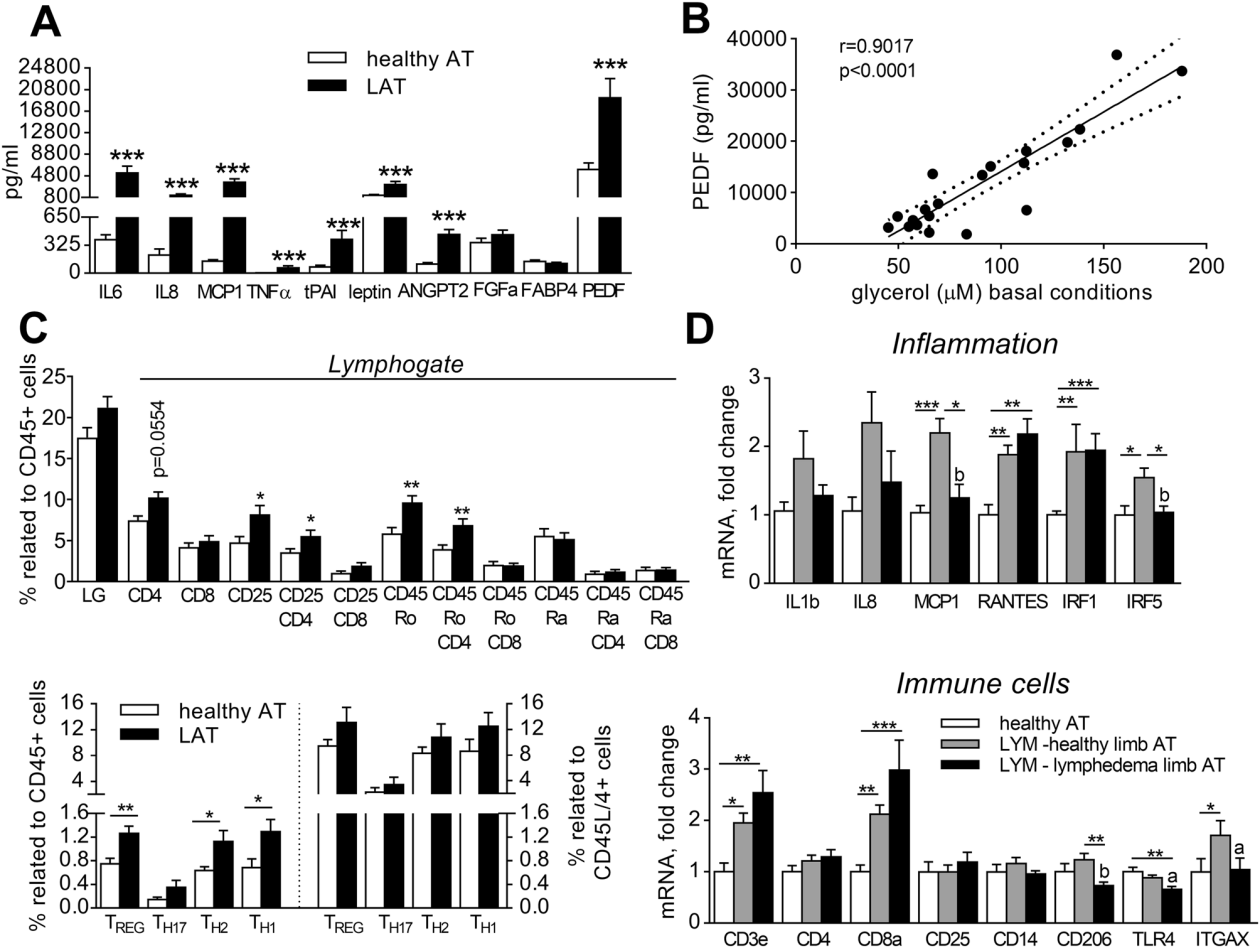
Immune parameters of LAT. (**A**) Cytokines secretion from AT explants (unpaired T- test of LN transformed data, ***p < 0.001). (**B**) Correlation of PEDF and glycerol release from AT explants (Pearson correlation coefficient). (**C**) Relative content of immune cell populations in AT, expressed as percentage of CD45+ positive cells or CD45/CD4+ positive cells within lymphogate (Mann Whitney test, *p < 0.05, **p < 0.01). (**D**) mRNA levels of immune markers in whole AT expressed as fold change over the mean expression of healthy group (Kruskal–Wallis test of 2^ΔCt^ values, Dunn’s correction, comparison of 3 groups, *p < 0.05, **p < 0.01, ***p < 0.001; Wilcoxon test of paired healthy and diseased limb of LYM subjects, a- p < 0.05, b- p < 0.01).

We first evaluated capacity of AT from lymphedema and healthy subjects to produce inflammatory cytokines ex vivo. Explants from LAT produced dramatically higher levels of inflammatory cytokines IL6, IL8, MCP1, TNFα, and tPAI (Fig. 3A). Also levels of leptin, ANGPT2 and PEDF were increased in media conditioned by LAT explants, while secretion of FGFacidic and FABP4 by AT was similar in both groups (Fig. 3A). Interestingly, PEDF levels correlated with the glycerol released by AT explants (Fig. 3B). EGF, VEGFA and VEGFC were not detectable in media conditioned by AT explants.

As production of cytokines by AT is partly dependent on infiltrated immune cells, immune cell content within SVF cells of AT was analysed by flow cytometry. Relative numbers of monocytes/macrophages and their phenotypic polarization in AT were not different between healthy and LYM group (Supplementary Fig. 1D). AT from lymphedema contained more CD45Ro expressing cells and activated CD4+ lymphocytes (CD4/CD25 +) including their subtypes (T_H_1, T_H_2, T_REG_, T_H_17) (Fig. 3C). However, when the distribution of CD4+ T cell subtypes was analysed within CD45/CD4+ cells, the difference between groups disappeared (Fig. 3C). Together, LAT contains more activated and memory T helper lymphocytes when compared with AT from healthy.

Markers of immune cells and inflammation were followed also on the level of mRNA isolated from intact AT. Expression of T lymphocyte markers CD3e and CD8a was higher in LAT compared to healthy AT, but there was no difference between healthy and diseased limb of LYM subjects. Also, no effect of the lymphedema was detected on the level of CD25. Markers of macrophages CD14, CD11c, (ITGAX) and CD206 were not different between healthy and LAT, while TLR4 expression was lower both in LYM group and in diseased arm of LYM subjects (Fig. 3D). Among analysed markers of inflammation, the expression of RANTES and IRF1 proved to be increased in LAT vs. healthy. But similarly as for T lymphocyte markers, there was no difference in the expression of the two genes between healthy and paired diseased limb of LYM subjects. Surprisingly, pro-inflammatory cytokine MCP1 and inflammation-related transcription factor IRF5 were more expressed in the healthy limb in LYM subjects compared to paired diseased limb, the same trend was observed for the expression of IL1β and IL8 genes (Fig. 3D). Gene expression data therefore did not match completely the findings based on the flow cytometry analysis and analysis of media conditioned by AT explants.

**Figure 4:**
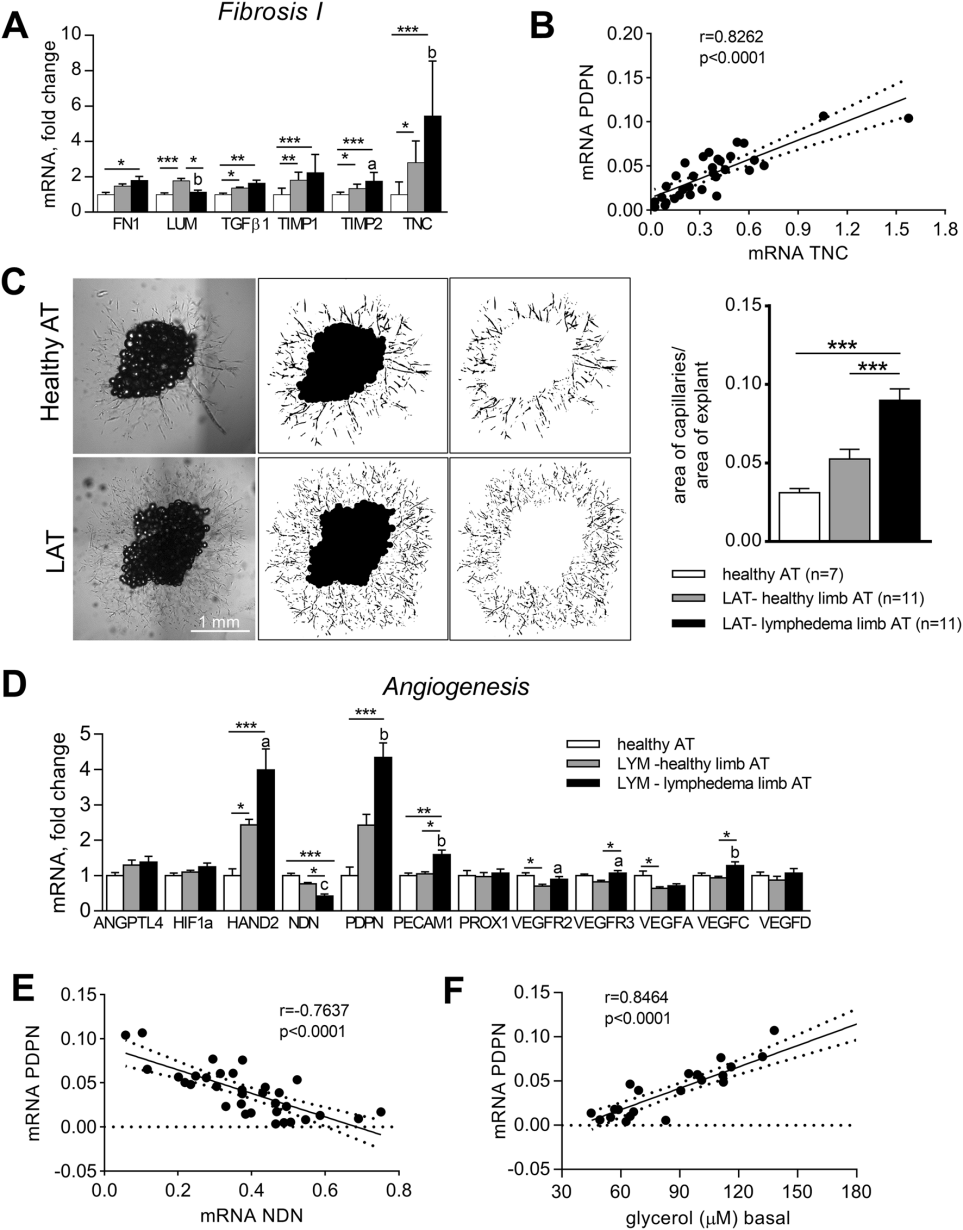
Fibrotic and angiogenic parameters of LAT. mRNA levels of fibrotic (**A**) and angiogenesis (**D**) markers for post-hoc analysis in whole AT expressed as fold change over the mean expression of healthy group (Kruskal–Wallis test of 2^ΔCt^ values, Dunn’s correction, comparison of 3 groups, *p < 0.05, **p < 0.01, ***p < 0.001; Wilcoxon test of paired healthy and diseased limb of LYM subjects, a- p < 0.05, b- p < 0.01, c- p<0.001). (**B**, **E**) Correlation of mRNA levels of PDPN with TNC (**B**) and NDN (**E**) in whole AT (Spearman correlation test). (**C**) Representative images of ex vivo angiogenic assay on AT explants with related image analysis (One way ANOVA of LN transformed data, Bonferroni correction for post-hoc analysis, comparison to diseased limb of LYM group, ***p < 0.001). (**F**) Correlation of mRNA PDPN with glycerol release in whole AT (Spearman correlation coefficient).

Chronic inflammation of any tissue is inevitably linked with fibrotic changes. Therefore, we assessed AT mRNA expression of genes associated with fibrosis. We have found that mRNA levels of TIMP2 and TNC were altered not only in LAT (when compared to healthy AT), but also in healthy vs. diseased limb within LYM group (Fig. 4A and Supplementary Fig. 1E). Expression of TNC correlated with mRNA of PDPN (Fig. 4B).

Inflammation is also a potent pro-angiogenic stimulus. To compare angiogenic potential of healthy AT and LAT, we set up ex vivo angiogenic assay according to Corvera’s group^15^ from explants derived from AT of healthy group and AT from both healthy and diseased limbs of LYM group. After 5 days of culture, area covered by capillary sprouting was much larger in LAT compared to healthy AT, when highest growth of capillaries was observed in AT from diseased limb (Fig. 4C). The cells from capillary sprouting were CD34 and CD31 positive (Supplementary Fig. 2).

Interestingly, in intact LAT there was a higher expression of mRNA HAND2, PDPN and PECAM, but lower expression of negative regulator of angiogenesis, NDN, compared to AT from healthy limb of LYM and healthy subjects (Fig. 4D). mRNA levels of all these genes were inter-correlated (Fig. 4E and Supplementary Table 2). In addition, upregulation of VEGFR2/3 and VEGFC in diseased vs. healthy limb was also observed (Fig. 4D). Notably, PDPN and NDN mRNA levels correlated with the area of capillary sprouting, but also with the expression of ATGL and PLINs, i.e. genes implicated in lipolysis, as well as basal glycerol and FFA levels released by AT explants (Fig. 4F, Supplementary Table 2). The latter finding indicate existence of a putative and so far underexplored functional connection between (lymphatic) endothelial cells and lipolysis.

### Characteristics of adipose progenitors in vivo and in vitro

Since reprogramming of adipose precursors by lymph stasis towards higher adipogenic potential could partially explain observed LAT expansion, we analysed numbers and behaviour of adipose precursors from both healthy and lymphedema AT.

**Figure 5:**
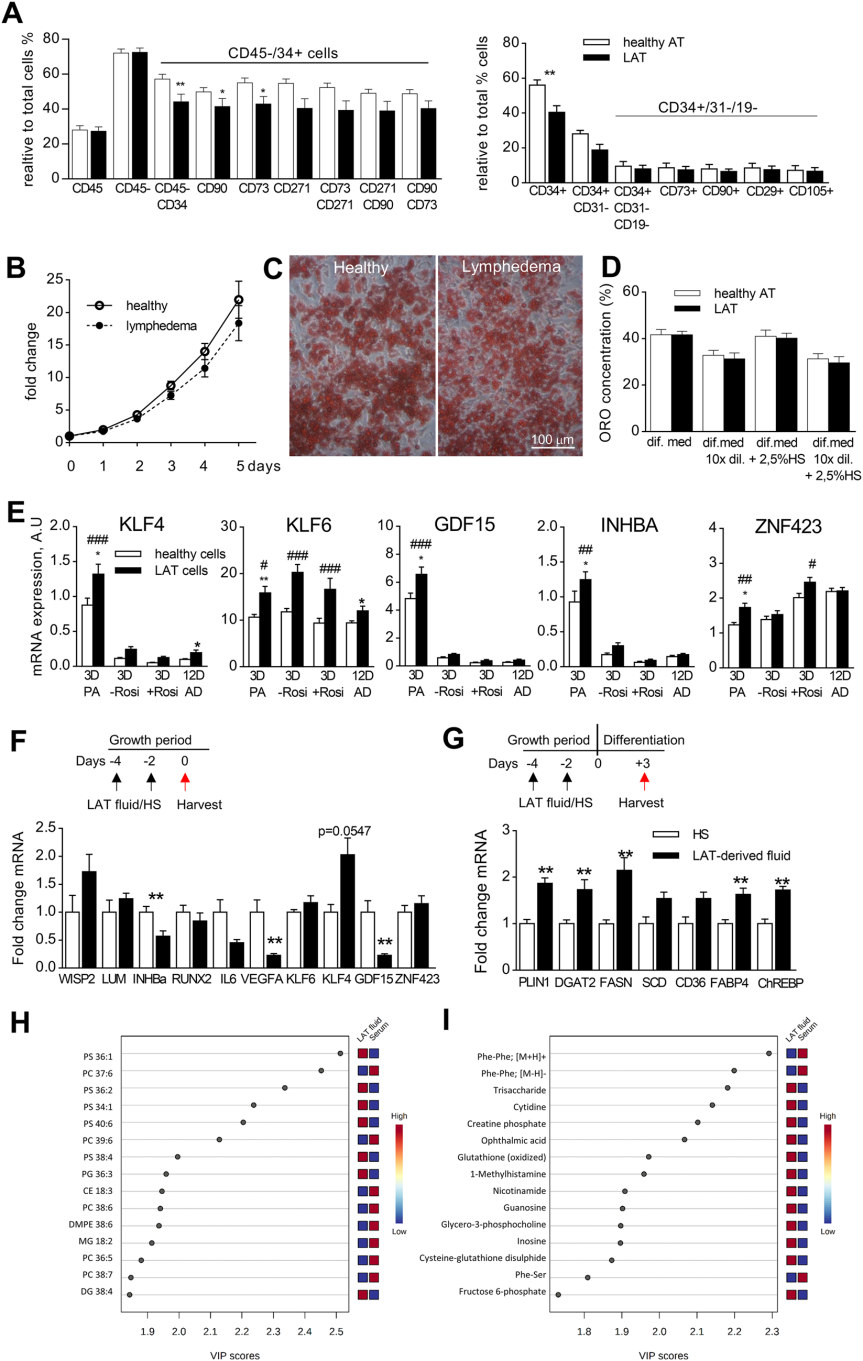
Characteristics of adipocyte precursors in LAT. (**A**) Relative content of mesenchymal stem cell populations in AT, expressed as percentage of total SVF cells (Mann Whitney test, *p < 0.05, **p < 0.01). (**B**) Wst1 assay on adipocyte precursors. Data are expressed as fold change of day 0 values (Two-way ANOVA, with Bonferroni correction for post-hoc analysis). (**C**) Representative images of in vitro differentiated adipocytes from healthy and LYM women stained with Oil Red O. (**D**) Quantification of neutral lipid accumulation by Oil Red O staining, results for 4 different adipogenic protocols are shown. HS, human serum; dil., diluted. (Mann Whitney test). (**E**) mRNA expression of selected genes in preadipocytes, early and mature adipocytes under in vitro conditions. Preadipocytes (PA, 3D) were harvested after 3 days in basal medium (serum free), early adipocytes after exposition to adipogenic medium (serum free) lacking or containing Rosiglitazone for 3 days (3D plus or minus Rosi) and mature adipocytes after completed adipogenic protocol (12 days, 12D AD) (Two-way ANOVA of 2^ΔCt^, Sidak post-hoc analysis, #p < 0.05, ## < 0.01, ###p < 0.001; Mann Whitney test of expression in preadipocytes and mature adipocytes analysed individually, *p < 0.05, **p < 0.01). (**F**,**G**) mRNA levels of selected markers in preadipocytes/early adipocytes exposed to LAT fluid or paired serum (HS). Data are expressed as fold change over the mean expression of cells cultivated in the presence of HS. Preadipocytes were either harvested immediately after 4 days in the presence of LAT fluid/ paired serum (**F**) or after additional 3 days when cells were induced to differentiate (early adipocytes, **G**) (Wilcoxon test, **p < 0.01). Variable importance in projection (VIP) plot with scores, to identify the top 15 most discriminating lipids (**H**) and polar metabolites (**I**). The coloured boxes indicate the relative concentrations of the corresponding metabolite in each group. CE: cholesteryl ester; DG: diacylglycerol; DMPE: dimethylphosphatidyletanolamine; MG: monoacylglycerol; PC: phosphatidylcholine; PS: phosphatidylserine.

First, we compared relative numbers of mesenchymal stem cells (MSC) in AT from healthy subjects and lymphedema patients by flow cytometry. Within CD45 negative population, relative content of MSC defined as CD34+ or CD31− was found to be lower in LAT. However, more detailed analysis of MSC utilizing combination of other MSC markers (CD90, CD73, CD271, CD29 and CD105) did not confirm these differences (Fig. 5A). Thus, abnormal AT accumulation in lymphedema cannot be explained by higher numbers of MSC in lymphedema patients.

Next, we analysed in vitro proliferation of MSC/preadipocytes isolated from AT of healthy and LYM subjects. Preadipocytes from LAT exhibited similar proliferation rate as cells from healthy subjects (Fig. 5B), also population doubling time at passage 3 and passage 10 was not different between the groups (passage 3, healthy AT- 40.6 ± 2.0 h, LAT 44.8 ± 3.7 h; passage 10, healthy AT 106.7 ± 19.0 h, LAT 131.2 ± 22.8 h). Thus, abnormal accumulation of AT in lymphedema is probably not caused by faster proliferation of adipose precursors that have comparable proliferative capacity to control cells at least when kept under standard in vitro conditions.

Therefore, we compared the adipogenic capacity of these precursors in response to four different media imitating optimal and suboptimal adipogenic conditions-standard serum free differentiation medium, serum free medium with 10 times lower concentration of components of hormonal mix (rosiglitazone, T3, dexamethasone, IBMX) and these two media supplemented with 2.5% human serum. Neutral lipid accumulation measured by Oil Red O assay was not different between the cells from lymphedema patients and healthy controls in any of the tested media (Fig. 5C,D). We also analysed the degree of differentiation at the mRNA expression levels of typical adipogenic markers (PPARγ, perilipin, FAS, DGAT2, ATGL) -but we could not find major differences between cells derived from lymphedema patients and control subjects (Supplementary Fig. 3E).

Nevertheless, preadipocytes derived from lymphedema subjects exhibited differential expression of GDF15, the marker of mitochondrial stress, and of KLF4, KLF6, INHBA and ZNF423, i.e. genes implicated in adipogenesis, when compared with cells from healthy women (Fig. 5E). Expression levels of KLF4 and 6 were correlated (Supplementary Fig. 3F). However, expression pattern of these genes did not clearly support the hypothesis that cells from lymphedema subjects have higher intrinsic adipogenic capacity (in other words, cells from lymphedema patients had higher expression of both pro- and anti-adipogenic markers). Expression patterns of remaining analysed genes was not different (Supplementary Fig. 3).

Also major metabolic functions of in vitro differentiated adipocytes including responsiveness to insulin (assessed as Akt phosphorylation and insulin antilipolytic action in Br-cAMP induced lipolysis) and degree of basal and stimulated lipolysis appeared to be independent on the cell origin (healthy vs. lymphedema AT) (Supplementary Fig. 1F,G). Together, abnormal AT accumulation in lymphedema cannot be explained by higher numbers of mesenchymal stem cells or better proliferative, adipogenic or metabolic potential of preadipocytes/adipocytes.

Thus, we expected that enhanced accumulation of lipids in adipocytes seen in lymphedema patients could be directly stimulated by undrained lymph/interstitial fluid. We succeeded to collect fluid from LAT of 6 lymphedema patients and this fluid was then used to supplement culture media, in parallel with paired serum (human serum, HS) from the same patients. Since the application of 10% LAT fluid together with adipogenic hormones caused cells to peel of the culture ware, we used LAT fluid or HS to supplement growth media of subconfluent preadipocytes. Medium was changed twice so the cells were exposed to LAT fluid/HS for 4 days. Then a part of the cells was harvested to assess the impact of LAT fluid on gene expression in preadipocytes, while remaining cells were induced to convert to adipocytes in serum free medium with 10 times diluted hormonal mix (suboptimal differentiation). After 3 days, cells were harvested and mRNA levels of adipogenic markers were analysed. In preadipocytes, we observed lower expression of INHBA, VEGFA and GDF15 in the presence of LAT fluid (Fig. 5F). In 3 day differentiated adipocytes, a majority of adipogenic markers was upregulated in the presence of LAT fluid compared to HS (Fig. 5G). Also, we monitored the effect of LAT fluid/HS on ongoing adipogenesis, i.e. LAT fluid/HS was applied on cells in day 6 of adipogenesis (for the growth of preadipocytes and then the induction of adipogenesis the standard conditions were used). In the middle of differentiation, LAT fluid had less intense effects, positively affecting only PLIN1 expression (not shown). To get insight into the differences between LAT fluid and serum, we analysed lipolytic products in these two fluids. We found that LAT fluid had higher concentration of glycerol and FFA than paired serum samples (glycerol: serum- 92.29 ± 14.96 µM, LAT fluid – 704.5 ± 54.65 µM; FFA: serum- 0.934 ± 0.135 mM, LAT fluid—2.542 ± 0.297 mM). Also, we performed untargeted metabolomic and lipidomic analysis of these two types of body fluids. Using four different gas chromatography–mass spectrometry platforms, we annotated 125 polar metabolites and 457 lipids (Supplementary Tables 4 and 5). 56 polar metabolites (45%) and 241 lipids (53%) were found to be differentially represented in LAT fluid vs. serum. Partial least squares discriminant analysis (PLS-DA) clearly separated LAT fluid and serum in case of both polar and nonpolar metabolites, indicating fundamental differences between the two body fluids (Suppl. Fig. 4A,B). Variable importance in projection (VIP) identified a group of phosphatidylserines (33%, higher content in LAT fluid vs. serum) and phosphatidylcholines (33%, higher content in serum vs. LAT fluid) among the top 15 most discriminating lipids (Fig. 5H). In case of polar metabolites, the highest VIP score was found for dipeptides (20%, higher content in serum vs. LAT fluid), nucleotides and glutathione related molecules (each 20%, higher content in LAT fluid vs. serum) (Fig. 5I). Majority of the differentially presented polar metabolites (53 out of 56) and 19% of lipids (45 out of 241) had higher content in LAT fluid vs. serum. In addition, the concentration of several cytokines was clearly different between the two fluids. The concentrations of sVEGFR3 and adiponectin were higher in plasma compared to LAT fluid, while the concentrations of leptin and IL6 were higher in LAT fluid vs. plasma (Supplementary Fig. 4C). Finally, concentration of all 8 analyzed miRNA species was several fold higher in LAT fluid than in serum (Supplementary Fig. 4D). These results suggest that undrained LAT fluid, rich in lipolytic products and miRNAs and with metabolome distinct from serum, may enhance the sensitivity of preadipocytes to adipogenic stimuli and thus represents an extrinsic proadipogenic factor acting mainly in the commitment step of adipogenesis.

## Discussion

Despite the fact that later stages of secondary lymphedema are associated with massive accumulation of AT, it is still rather obscure, what are the characteristics of this aberrant tissue. By combination of ex vivo and in vitro techniques, our study aimed to provide new insight into the properties of human lymphedema associated AT (as well as healthy AT from upper extremity) to provide basic clues necessary for future treatment of this aberrant AT accumulation.

Lymphedema-associated subcutaneous AT is rather insensitive to dietary intervention compared to healthy AT^16^ and surprisingly, despite its substantial amount (especially when the lower body is affected), fibrotic changes and signs of higher inflammation, its association with worsened metabolic health was not reported yet. The lack of lymphedema-impact on glucose and insulin levels and related HOMA-IR index was suggested also by the result of our study. Nevertheless, it appears that lymphedema is associated with higher relative content of lymphocytes, lower serum level of ZAG protein and altered concentration of several miRNAs in peripheral blood. While the cause and functional consequence of the observed lymphocytosis is not clear, ZAG is a cytokine produced by adipocytes and its levels were shown to be decreased under the conditions of zinc deficiency seen in obesity and other inflammatory disorders^17^. Zinc deficiency in lymphedema was suggested but not confirmed yet in clinical study. In addition, at least four of miRNAs found to be differentially expressed in serum of LYM subjects could regulate adipogenesis. While miR-29 and 144 were found to be positive regulators of adipogenesis (but their levels were decreased in LYM), miR-130b and 155 have the negative effect on differentiation of adipocytes in vitro^18^ (and their levels were increased in LYM). Thus, these circulating miRNAs in LYM subjects could limit rather than stimulate the expansion of AT^19,20^.

One of the findings of our study is the alteration of lipolysis and (lymph)angiogenesis in LAT. Higher capacity for (lymph)angiogenesis could be expected based on mRNA levels of several (lymph)angiogenic markers and results of ex vivo angiogenic assay. The most striking was higher expression of PDPN in AT from diseased limb of LYM subjects compared to healthy limb and healthy subjects. Expression of PDPN outside of kidneys is specific for lymphatic endothelial cells^21^ and also for stem cells that may serve as endothelial progenitors^22^. Also the expression of HAND2, the gene that has been not connected with lymphangiogenesis yet but was implicated in cardiac angiogenesis^23^, and NDN, a growth suppressor inhibiting angiogenesis^24^, were deregulated by lymphedema. The assumption that these genes are functionally linked to (lymph)angiogenesis is supported by the correlation of their expression with the ex vivo AT angiogenic capacity. Even though lymphatic vessels are rare within human subcutaneous AT^25^, our results suggest that LAT secretion (ANGPT2 and PEDF^26,27^) could potentiate excessive (lymph)angiogenesis (leading to the growth of dysfunctional vessels^28,29^) and thus further strengthen the vicious circle between lymph leakage and aberrant AT accumulation.

Interestingly, PEDF has been shown also as a potent activator of ATGL, one of the major lipases in AT^30,31^. This goes hand in hand with our data showing higher basal lipolysis in LAT and correlation between PEDF and glycerol levels in media conditioned by AT explants. Higher basal lipolysis in LAT was observed despite decreased mRNA levels of ATGL. Nevertheless, enzymatic activity of ATGL does not always correspond with its transcript levels^32^. Interestingly, expression of PLIN1 and 3 was increased in LAT. The correlation of the expression of ATGL/ PLINs with the expression of PDPN, together with the correlation between mRNA PDPN and basal glycerol release suggests an existence of a yet unknown crosstalk between lipolysis and lymphangiogenesis. This putative connection is of great interest since lymphatics can drain the products of lipolysis from AT^33^ and that lymphangiogenesis requires ongoing fatty acid oxidation^34^.

Another interesting and rather surprising result of our study was the observation of high FFA:glycerol ratio in media from LAT explants under basal conditions, which was comparable to the ratio of these lipolytic products upon adrenergic stimulation. In contrast, healthy upper limb AT exhibited extremely low FFA:glycerol ratio during basal lipolysis suggestive of either increased reesterification or catabolism of released FFA. As this result could not be replicated on isolated adipocytes, cells of SVF are probably involved in the metabolism of released FFA. It is feasible that immune cells in LAT produce more cytokines preventing the effective re-esterification under basal conditions. Indeed, higher secretion of IL6 by LAT could contribute to this phenomena^35^.

Murine model of lymphedema revealed a substantial increase in the numbers of CD4+ T lymphocytes and macrophages upon surgical damage to lymphatic system^11^. The increased infiltration of lymphedematous tissue by CD4+ T lymphocytes persisted for 6 weeks after the axillary lymph node dissection, while increased numbers of macrophages were marked for only 3 weeks but not in later stages. Similarly, in our study analysing samples from chronic stages of secondary lymphedema, we found higher relative content of activated CD4+ T lymphocytes but no significant increase in the content of macrophages when compared with healthy AT. The lack of higher infiltration of LAT by macrophages is also in line with the findings of the study by Tashiro et al.^13^, probably the only available human study describing immune cell content in LAT, that reported even lower number of CD45 hematopoietic cells and crown-like structures in lower extremity LAT compared to AT from the healthy limb. Concerning higher content of CD4+ T lymphocytes, it is however questionable whether the overall signalling originating from CD4+ T lymphocytes is substantially altered as the balance between the individual subtypes of CD4+ T lymphocytes was similar in both types of AT.

Besides of immune cells, expression of pro-inflammatory cytokines is another sign of tissue inflammation. In this case, we have proved much higher secretion of 5 pro-inflammatory cytokines from ex vivo cultivated LAT explants. The strikingly increased secretion of these pro-inflammatory cytokines was however in contrast with their almost unchanged mRNA levels – in fact, mRNA level of MCP1 was actually higher in healthy limb compared to diseased limb in LYM subjects and the similar trend was observed for IL8. No difference in mRNA levels of IL6 and IL8 between healthy and diseased limb AT of lymphedema patients were reported also in the first mRNA profiling study of AT from healthy and diseased limb of lymphedema patients^36^. Thus, higher secretion of pro-inflammatory cytokines from LAT may be a result of either strongly enhanced stability or translation of these mRNAs or mechanical release of cytokines from the interstitial space of AT where they may be “trapped”. Later hypothesis could be supported also by the fact that no difference was found in plasma levels of these cytokines that are partially dependent on the production from AT.

Enhanced fibrosis in human LAT has been shown previously using histological methods^13^ and by mRNA profiling in AT^36^. We were able to confirm higher mRNA levels of TNC found by Panck et al. TNC was found to promote the formation of poorly functional blood vessels via regulation of Wnt signalling^37^. Interestingly, TNC was found to be elevated in visceral (but not subcutaneous) AT in obese humans^38^, i.e. in the AT depot with intimate connection with intestinal lymphatics.

Our result that adipocytes from LAT are not hypertrophic when compared to cells from healthy AT goes against data from Prox1 ± and apelin KO mice^5,39^ and data from a sole human study analysing adipocyte size in LAT from lower limb^13^. Previously published results of human study were based on the histological analysis of LAT and paired healthy AT from 3 subjects. We analysed size of isolated adipocytes obtained by collagenase digestion. Although we cannot exclude the possibility that lymphedema adipocytes are more fragile after collagenase digestion compared to healthy adipocytes, we are quite confident in the validity of used technique^40^. Another possible source of bias is the fact we did not compare paired samples, i.e. an approach eliminating inter-individual variability. Nevertheless, it could be expected that hyperplastic, though yet unconfirmed, growth is also involved in LAT expansion and this could explain generally higher proportion of smaller adipocytes in LAT seen in this study. Thus, type of LAT expansion (hyperplastic vs. hypertrophic) needs to be further studied.

In the animal model of secondary lymphedema, enhanced activity of PPARγ, which is the master regulator of adipogenesis and lipogenesis in adipocytes, has been detected in subcutaneous area. Therefore, expansion of LAT could be driven through enhanced adipogenesis or lipogenesis. Nevertheless, expression pattern of analysed lipogenic markers was not substantially changed in diseased limb of LYM subjects compared to healthy, while the expression of both inhibitors (GOT2,WISP2, INHBA) and activators (ZNF423, KLF9) of adipogenesis was enhanced. Accordingly, previous mRNA profiling in AT by microarrays did not detect any difference in the expression of typical lipogenesis or adipogenesis markers in healthy compared to diseased limb of lymphedema patients^36^. Interestingly, AT expression of only 40 genes (out of 16 000 genes) was found to be modified by lymphedema^36^. Thus, posttranscriptional regulation is probably more involved in the maintenance of the diseased phenotype of lymphedema.

In vitro, cells isolated from LAT differed from healthy cells by the increased expression of 4 genes involved in the regulation of adipogenesis (both pro- and anti-adipogenic regulators) that however did not result in the increased ability to undergo adipogenesis in vitro. In fact, KLF4 and KLF6, expression of which was correlated, are transcription factors with the mixed role in the regulation of adipogenesis^41^. Moreover, activin /INHBA is expressed in AT mainly by preadipocytes and infiltrated macrophages and while it inhibits adipogenesis it promotes growth of preadipocytes^42^. Despite its increased expression in LAT cells, we did not prove higher proliferative activity of LAT preadipocytes in vitro^41^. Nevertheless, we have to admit that the donors of LAT cells were older and fattier compared to healthy donors and all these factors could negatively affect the adipogenic and proliferative capacity of cells (as shown before by us and others ^43–45^) and thus possible obscure differences between the cells. Despite this potential bias, in the view of clinically obvious aberrant expansion of LAT even in older women it is still very likely that extrinsic signals present in undrained lymph may play a significant role in LAT expansion.

Indeed, increased pro-adipogenic potential of LAT fluid during in vitro adipocyte differentiation was shown in this study. LAT fluid used in our experiments represents presumably a mixture of undrained lymph, interstitial fluid and content of cells damaged during the liposuction. Both the lymph and the interstitial fluid contain unique mixture of tissue specific metabolites, lipids, proteins and RNA molecules in different concentrations than found in plasma^33,46^. Thus, they may be a source of metabolites and signals that directly facilitate adipocyte proliferation and hypertrophy. It was shown before that lipid-rich chyle (lymph derived from intestines) promotes adipogenesis primarily via high content of particular fatty acids^47^. Although lymph originating from subcutaneous area does not contain chylomicrons representing the major source of lipids in chyle, LAT fluid contained almost 3 times more free fatty acids than serum. Some of these fatty acids could serve as ligands for PPARγ, as in our experiments LAT fluid acted mostly in the commitment/ early adipogenesis steps not in later steps of adipogenesis. It would fit well with the observation that the expansion of AT does not occur immediately when lymph stasis develops. Moreover, LC–MS analysis revealed substantial differences in the composition of lipidome/metabolome of LAT fluid and serum. Presence of phosphatidylserines and nucleotides (i.e. typical cellular components) in LAT fluid suggests LAT fluid carries also the markers of the cellular lysis similarly as documented for lymph^46^. Importantly, even though the cellular damage can be caused artificially by fluid acquisition, the levels of adiponectin (marker of adipocytes), which were lower in LAT fluid (similarly as in the afferent lymph^33^) than in serum, argue against the major contribution of liposuction- driven cellular damage to the LAT composition. Also additional signals as miRNAs could modulate the commitment of adipose progenitors^48^. Indeed, LAT fluid exhibited a high content of miRNAs. It remains to be determined whether the source of these miRNAs are adipocytes from LAT, but it has been shown already that AT substantially contributes to overall circulating miRNA levels in mice^49^.

Major limitation of the current study is rather small number of subjects that were included, which is, however, typical for hypothesis generating studies. Thus, our conclusions need to be validated in a larger confirmatory study. Also, some analyses of AT could not be performed on paired samples from healthy and diseased limb in LYM subjects- due to obvious ethical reason- samples from healthy limb were very small, enabling to perform only gene expression analysis and ex vivo angiogenic assay. Nevertheless it should be pointed out that the “healthy arm” of lymphedema patients also exerts signs of the dysfunction of lymphatic system^50^. Thus, the best comparison would be between LYM and NO LYM women. However, there was a strong ethical concern about acquisition of AT in the amount necessary for all performed experiments (approx. 10 g) from the ipsilateral limb of breast cancer survivors (NOLYM group). Therefore we are aware that the analyses not including either paired samples from LYM group or AT from NOLYM might be partially biased, since they cannot exclude the impact of either lower age and adiposity or cancer therapy on the followed variables. Also ex vivo analyses do not reproduce the real in vivo microenvironment, shown correlations do not provide cause and effect information and mRNA results were not confirmed on the protein level or enzymatic activity on majority of occasions. Nevertheless, despite all these limitations we believe that the complexity of our study partially outweighs its limitations.

## Conclusions

This study presents results of first complex analysis of LAT and healthy AT from upper extremity. Analysis of healthy upper limb AT brought an interesting result of high re-esterification/utilization of FFA under basal conditions, which has not been reported for other AT depots. Identified LAT alterations (higher expression of lymphangiogenic markers, angiogenic capacity, basal lipolysis and lower FFA reesterification) provide the basis for the formulation of the hypothesis that the lymphedema-affected AT is characteristic by fruitless effort to restore the lymphatic drainage and to limit further storage of lipids. Our data suggested also a novel functional connection between (lymph)angiogenesis and lipolysis and that one of the triggers leading to the AT accumulation in breast cancer-related lymphedema is present in undrained lymph/interstitial fluid. Nevertheless, these new hypotheses need to be validated in the upcoming studies.

## Materials and methods

### Subjects

3 groups of women were recruited with the aim of initial matching for weight, BMI, fat mass and age: (i) 11 women with unilateral lymphedema in upper extremity (non-pitting breast cancer related lymphedema)-LYM group; (ii) 11 healthy women never diagnosed with lymphedema and cancer undergoing elective (aesthetic) liposuction-Healthy group; (iii) 11 women-breast cancer survivors without lymphedema- NOLYM group. Women with active cancer, impairment of hemocoagulation, deep vein thrombosis and angiodysplasia were excluded. All women within LYM group had stage III non pitting breast cancer related lymphedema, affecting whole ipsilateral upper extremity (from the arm to the wrist and often also the hand and fingers). Lymphoscintigraphy (performed after injection of Tc99m-labeled filtered sulfur colloid intradermally 1–2 cm proximal to the metacarpophalangeal joint) showed no functional collectors, radiopharmaceutical was trapped in the site of the application except for the flow through small dermal lymphatic vessels and the dermal backflow. Women included into the group Healthy group were never diagnosed with lymphedema and cancer. Breast cancer therapy in NOLYM and LYM subjects is summarized in Supplementary Table 3.

Anthropometric measurements, blood and AT sampling were performed after overnight fasting. AT was acquired from healthy and LYM women only, AT sampling was not performed on NOLYM women from ethical reasons. Fat mass was assessed by bioimpedance (QuadScan 4000, Bodystat, Douglas, British Isles) and also calculated from weigh, BMI and age according to the previously described equation^51^.

### Chemicals

Culture media, PBS and gentamicin were from Lonza (Basel, Switzerland) and FBS qualified for MSC was from Thermo Fisher Scientific (Waltham, MA, USA). FGFβ and EGF were supplied by Immunotools (Friesoythe, Germany), rosiglitazone was provided by Cayman (Tallinn,Estonia). Other chemicals were from Sigma Aldrich (St. Louis,USA).

### AT and LAT fluid acquisition

AT was collected from dorsal part of the forearm by liposuction (diseased arm of lymphedema patients or healthy subjects, yield approx. 15 g of AT) or needle biopsy (healthy contralateral arm of lymphedema patients, yield approx. 500 mg of AT). Both liposuction and needle biopsy were performed under general anaesthesia, while the upper limb was under compression. The type of liposuction used in lymphedema patients was traditional “dry liposuction” when only the pressure was applied and no infiltration of the tissue by saline was performed. The pressure blocks the blood flow into the arm during the whole procedure, so the obtained tissue was only minimally contaminated by blood, despite no use of adrenaline. Thus LAT fluid originates solely from the patient. The infiltration of tissue with adrenaline (liposuction in healthy subjects) was postponed until the AT samples were obtained.

AT samples from diseased limb of lymphedema patients were briefly centrifuged (300 g) to separate tissue from LAT fluid, LAT fluid was collected, aliquoted and frozen in -80 °C. AT was washed in sterile saline and under aseptic conditions split to several aliquots. Two aliquots (150–200 mg) were snap frozen in liquid nitrogen for subsequent gene expression analysis. 1–2 g of tissue was dedicated to isolation of preadipocytes, 10 g of tissue was used for isolation of adipose and stromavascular cells –these were utilized in lipolytic experiments, assessment of adipocyte size and FACS analysis of cellular composition of stromavascular fraction. Remaining AT was used for set up of AT explants.

### Collagenase digestion of AT

Minced AT was digested with 1.5 volume of Collagenase NB 4 (300 units/mL; Serva, Heidelberg, Germany) in PBS/2% BSA for 1 h in 37 °C shaking water bath. Digested tissue was filtered through 250 µm sieves to remove undigested scrap and centrifuged at 300 g for 10 min. Mature adipocytes were gently aspirated by cut 1 ml tip and collected for adipocyte size analysis and lipolytic experiment. Infranatant was discarded and SVF cells were used for flow cytometry and in vitro culture as described below. Viability of the SVF cells was about 90%, as tested by propidium iodide staining and subsequent flow cytometry analysis.

### Adipocyte size assessment

Isolated adipocytes were washed two times with PBS, then gently mixed and 50 µl aliquot was mixed with 50 µl 1% BSA/PBS solution and stained with methylene blue. Adipocytes were then transferred to microscope slide and covered with plastic Thermanox #1.5 coverslips. Images were captured by inverted microscope Leica DMI 6000 CS with 10 × objective and monochrome fluorescence camera Leica DFC 350 FX via software Leica LAS AF 2.7.2. Acquisition area was chosen randomly over each sample. Due to field of view of 10 × dry objective (1.28 × 0.96 mm) matrix scan 2 × 3 fields of view was used to cover adequate area with adipocyte for their recognition and size measurement. For each subject 3 images were taken (on average 350 adipocytes / picture), which were analysed using freeware software CellProfiler^52^. Analysis of frequency distribution (%) of adipocytes in groups defined by diameter size (20–190 µm with bins width 20 µm) was then performed.

### Lipolysis assay

AT explants—Approximately 1 g of AT was cut into small pieces, washed two times in Krebs/Ringer phosphate buffer (pH 7.4) supplemented with 20 g/L of FFA free BSA and 1 g/L of glucose (KRB). Then explants were incubated in 1.5 ml of KRB for 1 h at 37 °C in a shaking water bath and washed again to remove proteins from damaged cells. Then 100 mg of briefly dried AT per tube was carefully weighted and incubated in KRB supplemented with water or 1 µM isoproterenol to induce lipolysis. After 4 h of incubation at 37 °C in a shaking water bath, the conditioned medium was collected, the cellular debris was removed by centrifugation and cell-free supernatants as well as original AT tissue were stored at -80 °C until analysis. Lipid content in AT was assessed according to Dole. Briefly, 200 μL of PBS was added to the 100 mg tissue sample and sonicated using UP200S (Hielsher Ultrasound Technology, Germany) on ice. The sample was then mixed with 1.5 mL Dole’s extraction mixture (80% Isopropanol; 20% Heptane; 20 mM H_2_SO_4_), and subsequently with 900 μL Heptane and 900 μL H_2_O. After centrifugation, fatty phase was transferred to preweighed glass tube and the remnants of non-lipid phase were evaporated overnight in 70 °C. Lipids were weighed on the analytical balance next day.

Mature adipocytes–isolated adipocytes were washed two times with PBS. Then packed adipocytes were divided into 100 µl aliquots and mixed with 200ul KRB supplemented with 1 mU/ml adenosine deaminase and either water or 1 µM isoproterenol. Then adipocytes were incubated for 2 h in at 37 °C in a shaking water bath. Lipolysis was stopped by placing the tubes on ice, medium (infranatant) was collected after brief centrifugation and stored at -80 °C.

Adipocytes differentiated in vitro- 12 day differentiated adipocytes were washed twice with DPBS+ Mg^2+^ and Ca^2+^ and insulin starved for 18 h. Then they were washed once and treated with basal medium containing 0.5% FFA free, endotoxin low BSA and transferin supplemented with either vehicle (dH_2_O), 100 nM insulin, 1 µM isoproterenol or 1 mM 8bromo-cAMP or combination of 100 nM insulin and 1 mM 8bromo-cAMP for 3 h. Concentration of glycerol released into the media was normalized to protein content of cells lysed by 0,05 N NaOH.

### Plasma and conditioned media analyses

Levels of glucose, insulin, and lipid parameters were determined using standard methods in certified laboratories. FFA and glycerol levels were measured using enzymatic colorimetric kits (Randox, Crumlin, United Kingdom). Cytokines in plasma, LAT fluid and conditioned media were assessed by ELISA (Duosets, R&D Systems, Abingdon, United Kingdom) and/or Luminex (High-Sensitivity T cell panel, Merck-Millipore, Germany and Human Magnetic Luminex Assay, Bio-Techne, Minneapolis, MN, USA).

### Flow cytometry

Blood staining—50 µl of well mixed whole blood was incubated with appropriate monoclonal fluorescent labelled antibodies for 25 min in dark chamber, at room temperature. Erythrocytes were lysed with 2 ml of Erythrocyte lysis buffer (ELB; 0,1 mM EDTA, 0,15 M HN_4_Cl, 5,6 mM K_2_HPO_4_) for 15 min in dark, at room temperature. Cells were then centrifuged at 300 g for 5 min and washed with PBS. Pellet was resuspended in PBS and analysed immediately on BD FACSVerse (Becton, Dickinson and Company, Franklin Lakes, NJ, USA).

Stromavascular fraction (SVF) cell staining- SVF cells in pellet were diluted in 5 ml of ELB, incubated at room temperature for 15 min and then filtered through 100 and 40-µm sieves to get single cell suspension. Cells were then collected by centrifugation at 300 g for 10 min. Pellet was diluted in 1 × PBS/0,5% BSA/2 mM EDTA to get final concentration 10^6^ cells /ml. Calculation was made by using Burker´s chamber. Cells were stained with appropriate monoclonal fluorescent labeled antibodies in 4 °C, at dark for 25 min and analysed immediately by BD FACSVerse.

Antibodies were from BD Biosciences (San Jose, CA, USA):CD45 PerCP clone 2D, CD14 FITC clone M5E2, CD40 PE-Cy7 clone 5C3, CD282 AF 647 clone 11G7, CD206 BV 421 clone 19.2, CD206 APC clone 19.2, CD16 APC-Cy7 clone 3G8, CD4 FITC RPAT4, CD3 PE clone UCHT1, CD8 Alexa 647 RPAT8, CD45Ro APC H7 clone UCHL1, CD45Ra BV510 clone HI100, CD11c BV510 clone B-ly6, CD127 PE clone hIL-7R-M21, CD25 PE-Cy7 clone M-A251, CD196 APC clone 11A9, CD183 BV421 clone 1C6/CXCR3, CD194 BV510 clone 1G1, CD90 FITC clone 5E10, CD73 APC clone AD2, CD271 BV421 clone C40-1457, CD105 PerCP-Cy5.5 clone 266, CD31 APC-Cy7 clone WM59, CD19 BV421 clone HIB19, CD29 BV510 clone MAR4; Biolegend (San Diego, CA, USA): CD284 PE clone HTA125, Exbio (Vestec, Czech Republic): CD163 PE clone GHI/61, CD11c Pacific Blue clone Bu15, CD146 PE clone P1H12, CD34 PE-Cy7 clone 4H11 [APG]; Miltenyi Biotec (Bergisch Gladbach, Germany): CCR2 Pe-Vio770 clone REA264. The gating strategy was similar to that described by Cizkova et al.^53^.

### Isolation, culture and differentiation of preadipocytes

Isolation, culture and differentiation of preadipocytes was described previously^43^. Briefly, SVF cells were incubated in ELB for 10 min, RT, then filtered through 40um sieve and collected by centrifugation. The supernatant was carefully discarded and cells were resuspended in the growth medium (PM4) and plated into 35 mm dish. Cells were subcultivated upon 70% confluence and passed twice prior cryopreservation. For experiments, cells were plated in the density 10 000 cells/cm^2^ and allowed to grow until they were 2 days postconfluent. Differentiation was then induced by DMEM/F12 medium supplemented with 66 nM insulin, 1 µM dexamethasone, 1 nM T3, 10 µg/ml transferin, 0.25 mM IBMX, 1 µM rosiglitazone. After 6 days rosiglitazone and IBMX were omitted and dexamethasone was replaced with 0.1 µM cortisol. The differentiation continued until day 12. In certain experiments, differentiation medium was supplemented with 2.5% FBS or 2.5% human serum (HS) collected from 3 healthy human volunteers.

When effect of LAT fluid on adipogenesis was tested, LAT fluid and paired HS was applied on standard culture of gluteal preadipocytes (mixed cells from 5 healthy obese women).This experiment was repeated twice, with samples from six LYM women.

### Oil Red O (ORO) staining

12 days differentiated cells were fixed and stained as described previously^43^.

### WST-1 assay

Preadipocytes were seeded at the density of 4000 cells/cm2 in 100 µl PM4/well in 96- well plate in quadruplicates. After 5 h of seeding, 10 µl of Wst-1 reagent was added into each well. Formazan formation was measured after 2 h at 440 nm on spectrophotometer (Versamax, Molecular Devices, San Jose, CA, USA) and subsequently after every 24 hours for 5 consecutive days.

### Western blotting

Adipocytes were washed two times with PBS and lysed on ice for 15 min in RIPA lysis buffer supplemented with protease and phosphatase inhibitors (Complete, PhoStop, Roche Diagnostics, Mannheim, Germany). Lysates were frozen in liquid nitrogen and let thawed on ice then centrifuged for 15 min at 15 000 g, 4 °C. Protein concentration was determined using the bicinchoninic assay, Pierce (Rockford, IL, USA). Samples were loaded to a 10% acrylamide minigels and electrotransferred onto the nitrocellulose membranes. Membranes were blocked with 5% BSA. Antibodies against Akt and its phosphorylated form were from Cell Signaling (Danvers, MA, USA). Antigen–antibody complexes were detected using secondary antibodies coupled with horseradish peroxidase and the ECL detection system (Pierce) in Carestream Gel Logic 4000 PRO Imaging System equipped with Molecular Imaging Software (Carestream Health, New Haven, CT, USA).

### Angiogenic assay

AT was cleaned and cut into small pieces approximately 1 mm^3^ that were carefully positioned into 38 µl Matrigel (Matrix Basement Membrane Growth Factor Reduced, Corning, Bedford, USA) per well in 96-well plate. For each patient 24 AT explants from lymphedema limb and 24 AT explants from paired healthy limb were set up. The plate was placed into the incubator to polymerize for 30 min at 37 °C, then 200 µl of supplemented EGM-2 MV media (Lonza) was added into each well and incubated at 37 °C in 5% CO_2_ atmosphere. 100 µl of EGM-2 MV media was replaced every second day. Analysis of endothelial outgrowths was performed at day 5. Inverted fully automated research microscope Leica DMI 6000B with attached highly sensitive monochrome fluorescence camera Leica DFC 350 FX R2 were used for image acquisition. 96 well plate was inserted into EMBL climate chamber (EMBL Heidelberg) preheated to 37 °C in the scanning stage. Images were recorded in bright field mode with dry lens Leica HCX PL Fluotar 5x/0,15 in single or matrix up to 2 × 2 fields TileScan mode. Analysis of acquired data was realized by open-source software for biological analysis Fiji^54^. Written macro allows automatic recognition within single field of view samples with homogenous illumination and minimum amount of artefacts. In case of matrix scans, the macro was adapted for user setting of threshold to omit external artefacts.

### Gene expression analysis

Total RNA from AT and cells was isolated using miRNeasy and RNeasy Mini Kit, resp. (Qiagen, Germany). RNA concentration was measured using Nanodrop1000 (Thermo Fisher Scientific, USA). DNAse I (Thermo Fisher Scientific, USA) treatment was applied to remove genomic DNA. Four to six hundred nanograms of total RNA were reverse transcribed using a high-capacity cDNA reverse transcription kit (Applied Biosystems) as described previously^55^. For microfluidic real-time quantitative PCR, 4 ng of cDNA was preamplified within 16 cycles (TaqMan Pre Amp master mix kit; Applied Biosystems). For the preamplification, 20 × TaqMan gene expression assays of all target genes (the list of genes in Supplementary Table 1) were pooled together and diluted with water to the final concentration 0.2 × for each probe. The real-time qPCR was performed in duplicates on Biomark real-time qPCR system using 96 × 96 array as a paid service. Data were normalized to the arithmetic mean of two reference genes: TBP and RPS13, which proved to be superior over two other measured reference genes, PPIA and GUSB (not shown). Preamplification of mRNA FABP4, ADIPOQ and ACLY in in vitro differentiated adipocytes resulted in very low Ct and therefore these results were not included into analysis.

### miRNA analysis

Only serum samples without haemolysis detected spectroscopically (method evaluating presence of haemoglobin as described by Harboe-the formula used to calculate hemoglobin concentration was as follows (Hb (g/l) = (k[167·2 × A415 − 83·6 × A380 − 83·6 × A450]) ÷ 1000)) was used to isolate miRNA. miRNA was extracted from 250 µl of serum or LAT fluid spiked with 1 pM of cel-miR-39 using mirVana PARIS RNA isolation system. miRNA samples were diluted two times with dH_2_0 prior reverse transcription. cDNA synthesis was performed by miRCURY LNA RT kit (Qiagen) according to the manufacturer’s recommendations in the presence of Unisp6 RNA spike. cDNA was diluted 1:10 with dH_2_O just before qPCR utilizing miRCURY miRNA Focus panel PCR Panel PLASMA or miRCURY LNA miRNA PCR Assay (YP00204243, YP00204499, YP00204679, YP00205892, YP00204317, YP00205911, YP00204260), miRCURY LNA SYBR Green PCR kit and ABI 7500 Fast instrument. Individual runs of miRCURY Focus panels were normalized to Unisp6spike, samples found to be haemolytic according to the delta Ct between of miR23a and miR-451 higher than 7 were excluded. Thus, LYM group was represented by 8 samples and the group of nonlymphedema subjects (including healthy and NOLYM women, which were combined to reach higher statistical power for testing the hypothesis) were represented by 7 samples. Obtained Ct numbers were analysed by GenEx software 7.0 (MultiD, Goteborg, Sweden), 8 miRNA were excluded from analysis because of Ct value higher than 35. NormFinder algorithm was used to select endogenous controls (suitable endogenous control for comparison of miRNA levels in LAT fluid and serum was not found). Dynamic PCA was applied on selected miRNA species.

### LC–MS analysis

Extraction of serum and LAT fluid and following LC–MS analysis was performed as described in Brezinova et al.^56^.

### Statistical analysis

GraphPad Prism 6.0 software was used for data analysis. To analyse differences between groups or treatments, Wilcoxon, Mann–Whitney, 2 way ANOVA, Kruskal Wallis or unpaired t-tests were performed as appropriate (the used statistical test is stated in the Figure legend). Data in Table 1 are presented as mean ± SD, all other data are presented as mean ± SEM. Correlations of gene expression data (from both experimental groups together) were performed using Spearman’s test. The LC–MS data were processed and analyzed by MetaboAnalyst version 5.0^57^. The level of significance was set at p ≤ 0.05.

### Ethics approval and consent to participate

All procedures on human volunteers were in accordance with the 1964 Helsinki declaration and approved by ethics committee of the Kralovske Vinohrady University Hospital. Informed consent was obtained from all participants included in the study.

## Supplementary Information

Supplementary Information 1.

Supplementary Information 2.

Supplementary Information 3.

Supplementary Information 4.

Supplementary Information 5.

Supplementary Information 6.

Supplementary Figure 1: Effects of lymphedema on systemic variables and AT qualities. (A) The 2D score plot of the PCA of miRNA expression that characterizes the trends exhibited by the expression profiles of LYM (red) and non-lymphedema subjects (combined heathy and NOLYM, green). Each dot represents a subject. The degree of variability is expressed as a percentage. (B) Distribution of adipocyte size. Histogram of frequencies of cells within 20 µm bins (Two-way ANOVA, Sidak post-hoc analysis, *p <0.05). (C) Ex vivo lipolysis in isolated adipocytes. Concentrations of glycerol and FFA normalized to mg of lipids as well as fold change over the basal conditions are shown. ISO-1µM isoproterenol, ADA, adenosine deaminase (Two-way ANOVA of LN transformed data, Sidak post-hoc analysis). (D) Relative content of macrophage populations in AT, expressed as percentage of CD45+ positive cells (Mann Whitney test). (E) mRNA levels in whole AT expressed as fold change over the mean expression of healthy group (Kruskal-Wallis test of 2^ΔCt^ values, Dunn’s correction, comparison of 3 groups; Wilcoxon test of paired healthy and diseased limb of LYM subjects).(F) Phosphorylation of Akt in lysates from adipocytes exposed to insulin for 5 or 20 minutes. Signal for p-Akt antibody was normalized to total Akt signal. Ins, 100 nM insulin. (G) Analysis of in vitro lipolysis in adipocytes. Cells were exposed to basal conditions, isoproterenol, 8-Br-cAMP or insulin for 3 hours. ISO-1µM isoproterenol, cAMP, 1 mM 8-Br-cAMP, INS, 1nM insulin (Two-way ANOVA of LN transformed data, Sidak post-hoc analysis).

Supplementary Figure 2: Flow cytometry analysis of cells originating from endothelial sprouts. Cells were extracted from Matrigel upon the termination of the angiogenic assay and subcultivated in EGM2 medium. Then they were detached from the plastic culture ware by trypsinization, collected, stained for CD31 and CD34 and analysed by flow cytometry. The representative histograms showing the negative controls (unstained cells, black line) and stained cells (red line) are shown.

Supplementary Figure 3: mRNA expression of selected genes in preadipocytes, early and mature adipocytes under in vitro conditions. Preadipocytes (PA, 3D) were harvested after 3 days in basal medium (serum free), early adipocytes after exposition to adipogenic medium (serum free) lacking or containing Rosiglitazone for 3 days (3D plus or minus Rosi) and mature adipocytes after completed adipogenic protocol (12 days, 12D AD). (A) Angiogenic markers; (B) Markers of fibrosis and extracellular matrix (ECM); (C) Various; (D) Mitochondria-related genes; (E) Markers of lipogenesis and adipogenesis. Expression levels were calculated as 2^Δ^^Ct^ (Two-way ANOVA, Sidak post-hoc analysis, #p<0.05, ###p<0.001; Mann Whitney test of expression in preadipocytes and mature adipocytes analysed individually, *p <0.05). F. Correlation between mRNA levels of KLF4 and KLF6 in preadipocytes (Spearman correlation coefficient).

Supplementary Figure 4: Comparison of composition of LAT fluid vs. paired serum. PLS-DA score scatter plot of the identified lipids (A) and polar metabolites (B) reveals a clear separation between serum and LAT fluid (n=6); (C) Cytokine levels, measured by ELISA (n=6, paired t test, *p <0.05, *** p<0.001, ND-not detectable); (D) miRNA levels measured by qPCR (n=4). miR-16-5p, miR-24-3p and miR-15-5p were originally intended as endogenous normalizers, since they have stable expression in human serum, but they could not be used for normalization of miRNAs of interest in both LAT fluid and serum due to their substantially higher expression in LAT.

## Data Availability

The datasets generated during and/or analysed during the current study are available from the corresponding author on reasonable request.

## Abbreviations

AT: Adipose tissue
BMI: Body mass index
FFA: Free fatty acids
HDL: High-density lipoprotein
HOMA-IR: Homeostatic Model Assessment for Insulin Resistance
HS: Human serum
ISO: Isoproterenol
LAT: Lymphedema-associated adipose tissue
LDL: Low-density lipoprotein
LYM group: Women with unilateral lymphedema stage II in upper extremity
MSC: Mesenchymal stem cells
NOLYM group: Women-breast cancer survivors-without unilateral lymphedema
ORO: Oil Red O staining
PCA: Principal component analysis
SVF: Stromal-vascular fraction

## References

[1] Brorson H, Ohlin K, Olsson G, Karlsson MK (2009). Breast cancer-related chronic arm lymphedema is associated with excess adipose and muscle tissue. Lymphat. Res. Biol..

[2] Mehrara BJ, Greene AK (2014). Lymphedema and obesity: is there a link?. Plast. Reconstr. Surg..

[3] Szolnoky G, Dobozy A, Kemeny L (2014). Towards an effective management of chronic lymphedema. Clin. Dermatol..

[4] Karkkainen MJ, Saaristo A, Jussila L, Karila KA, Lawrence EC, Pajusola K, Bueler H, Eichmann A, Kauppinen R, Kettunen MI (2001). A model for gene therapy of human hereditary lymphedema. Proc. Natl. Acad. Sci. USA.

[5] Harvey NL, Srinivasan RS, Dillard ME, Johnson NC, Witte MH, Boyd K, Sleeman MW, Oliver G (2005). Lymphatic vascular defects promoted by Prox1 haploinsufficiency cause adult-onset obesity. Nat. Genet..

[6] Li Y, Zhu W, Zuo L, Shen B (2016). The role of the mesentery in Crohn's disease: the contributions of nerves, vessels, lymphatics, and fat to the pathogenesis and disease course. Inflamm. Bowel Dis..

[7] Nougues J, Reyne Y, Dulor JP (1988). Differentiation of rabbit adipocyte precursors in primary culture. Int J Obes.

[8] Aschen S, Zampell JC, Elhadad S, Weitman E, De Brot M, Mehrara BJ (2012). Regulation of adipogenesis by lymphatic fluid stasis: part II. Expression of adipose differentiation genes. Plast. Reconstr. Surg..

[9] Zampell JC, Aschen S, Weitman ES, Yan A, Elhadad S, De Brot M, Mehrara BJ (2012). Regulation of adipogenesis by lymphatic fluid stasis: part I Adipogenesis, fibrosis, and inflammation. Plast. Reconstr. Surg..

[10] Levi B, Glotzbach JP, Sorkin M, Hyun J, Januszyk M, Wan DC, Li S, Nelson ER, Longaker MT, Gurtner GC (2013). Molecular analysis and differentiation capacity of adipose-derived stem cells from lymphedema tissue. Plast. Reconstr. Surg..

[11] Zampell JC, Yan A, Elhadad S, Avraham T, Weitman E, Mehrara BJ (2012). CD4(+) cells regulate fibrosis and lymphangiogenesis in response to lymphatic fluid stasis. PLoS ONE.

[12] Rutkowski JM, Moya M, Johannes J, Goldman J, Swartz MA (2006). Secondary lymphedema in the mouse tail: Lymphatic hyperplasia, VEGF-C upregulation, and the protective role of MMP-9. Microvasc. Res..

[13] Tashiro K, Feng J, Wu SH, Mashiko T, Kanayama K, Narushima M, Uda H, Miyamoto S, Koshima I, Yoshimura K (2016). Pathological changes of adipose tissue in secondary lymphedema. Br. J. Dermatol..

[14] Ly CL, Kataru RP, Mehrara BJ (2017). Inflammatory manifestations of lymphedema. Int. J. Mol. Sci..

[15] Rojas-Rodriguez R, Gealekman O, Kruse ME, Rosenthal B, Rao K, Min S, Bellve KD, Lifshitz LM, Corvera S (2014). Adipose tissue angiogenesis assay. Methods Enzymol..

[16] Schmitz KH, Troxel AB, Dean LT, DeMichele A, Brown JC, Sturgeon K, Zhang Z, Evangelisti M, Spinelli B, Kallan MJ (2019). Effect of home-based exercise and weight loss programs on breast cancer-related lymphedema outcomes among overweight breast cancer survivors: The WISER Survivor Randomized Clinical Trial. JAMA Oncol.

[17] Severo JS, Morais JBS, Beserra JB, Dos Santos LR, de Sousa Melo SR, de Sousa GS, de Matos Neto EM, Henriques GS, do Nascimento Marreiro D (2020). Role of zinc in zinc-alpha2-glycoprotein metabolism in obesity: a review of literature. Biol. Trace. Elem. Res..

[18] Haider N, Larose L (2019). Harnessing adipogenesis to prevent obesity. Adipocyte.

[19] Martin EC, Qureshi AT, Llamas CB, Burow ME, King AG, Lee OC, Dasa V, Freitas MA, Forsberg JA, Elster EA (2018). Mirna biogenesis pathway is differentially regulated during adipose derived stromal/stem cell differentiation. Adipocyte.

[20] Chen Z, Lai TC, Jan YH, Lin FM, Wang WC, Xiao H, Wang YT, Sun W, Cui X, Li YS (2013). Hypoxia-responsive miRNAs target argonaute 1 to promote angiogenesis. J. Clin. Invest..

[21] Jha SK, Rauniyar K, Jeltsch M (2018). Key molecules in lymphatic development, function, and identification. Ann. Anat..

[22] Conrad C, Niess H, Huss R, Huber S, von Luettichau I, Nelson PJ, Ott HC, Jauch KW, Bruns CJ (2009). Multipotent mesenchymal stem cells acquire a lymphendothelial phenotype and enhance lymphatic regeneration in vivo. Circulation.

[23] Hamik A, Wang B, Jain MK (2006). Transcriptional regulators of angiogenesis. Arterioscler. Thromb. Vasc. Biol..

[24] Moon HE, Ahn MY, Park JA, Min KJ, Kwon YW, Kim KW (2005). Negative regulation of hypoxia inducible factor-1alpha by necdin. FEBS Lett..

[25] Redondo PAG, Gubert F, Zaverucha-do-Valle C, Dutra TPP, Ayres-Silva JP, Fernandes N, de Souza AAP, Loizidou M, Takiya CM, Rossi MID (2020). Lymphatic vessels in human adipose tissue. Cell Tissue Res..

[26] Souma T, Thomson BR, Heinen S, Carota IA, Yamaguchi S, Onay T, Liu P, Ghosh AK, Li C, Eremina V (2018). Context-dependent functions of angiopoietin 2 are determined by the endothelial phosphatase VEPTP. Proc. Natl. Acad. Sci. USA.

[27] Halin S, Rudolfsson SH, Doll JA, Crawford SE, Wikstrom P, Bergh A (2010). Pigment epithelium-derived factor stimulates tumor macrophage recruitment and is downregulated by the prostate tumor microenvironment. Neoplasia.

[28] Yuan Y, Arcucci V, Levy SM, Achen MG (2019). Modulation of immunity by lymphatic dysfunction in lymphedema. Front. Immunol..

[29] Ogata F, Fujiu K, Matsumoto S, Nakayama Y, Shibata M, Oike Y, Koshima I, Watabe T, Nagai R, Manabe I (2016). Excess lymphangiogenesis cooperatively induced by macrophages and CD4(+) T cells drives the pathogenesis of lymphedema. J. Invest. Dermatol..

[30] Borg ML, Andrews ZB, Duh EJ, Zechner R, Meikle PJ, Watt MJ (2011). Pigment epithelium-derived factor regulates lipid metabolism via adipose triglyceride lipase. Diabetes.

[31] Notari L, Baladron V, Aroca-Aguilar JD, Balko N, Heredia R, Meyer C, Notario PM, Saravanamuthu S, Nueda ML, Sanchez-Sanchez F (2006). Identification of a lipase-linked cell membrane receptor for pigment epithelium-derived factor. J. Biol. Chem..

[32] Zechner R, Zimmermann R, Eichmann TO, Kohlwein SD, Haemmerle G, Lass A, Madeo F (2012). FAT SIGNALS–lipases and lipolysis in lipid metabolism and signaling. Cell Metab..

[33] Miller NE, Michel CC, Nanjee MN, Olszewski WL, Miller IP, Hazell M, Olivecrona G, Sutton P, Humphreys SM, Frayn KN (2011). Secretion of adipokines by human adipose tissue in vivo: partitioning between capillary and lymphatic transport. Am. J. Physiol. Endocrinol. Metab..

[34] Wong BW, Wang X, Zecchin A, Thienpont B, Cornelissen I, Kalucka J, Garcia-Caballero M, Missiaen R, Huang H, Bruning U (2017). The role of fatty acid beta-oxidation in lymphangiogenesis. Nature.

[35] van Hall G, Steensberg A, Sacchetti M, Fischer C, Keller C, Schjerling P, Hiscock N, Moller K, Saltin B, Febbraio MA (2003). Interleukin-6 stimulates lipolysis and fat oxidation in humans. J. Clin. Endocrinol. Metab..

[36] Planck T, Parikh H, Brorson H, Martensson T, Asman P, Groop L, Hallengren B, Lantz M (2011). Gene expression in Graves' ophthalmopathy and arm lymphedema: similarities and differences. Thyroid: Off J. Am. Thyroid Assoc..

[37] Saupe F, Schwenzer A, Jia Y, Gasser I, Spenle C, Langlois B, Kammerer M, Lefebvre O, Hlushchuk R, Rupp T (2013). Tenascin-C downregulates wnt inhibitor dickkopf-1, promoting tumorigenesis in a neuroendocrine tumor model. Cell Rep..

[38] Catalan V, Gomez-Ambrosi J, Rodriguez A, Ramirez B, Rotellar F, Valenti V, Silva C, Gil MJ, Salvador J, Fruhbeck G (2012). Increased tenascin C and Toll-like receptor 4 levels in visceral adipose tissue as a link between inflammation and extracellular matrix remodeling in obesity. J. Clin. Endocrinol. Metab..

[39] Sawane M, Kajiya K, Kidoya H, Takagi M, Muramatsu F, Takakura N (2013). Apelin inhibits diet-induced obesity by enhancing lymphatic and blood vessel integrity. Diabetes.

[40] Laforest S, Michaud A, Paris G, Pelletier M, Vidal H, Géloën A, Tchernof A (2017). Comparative analysis of three human adipocyte size measurement methods and their relevance for cardiometabolic risk. Obesity.

[41] Hsieh PN, Fan L, Sweet DR, Jain MK (2019). The Kruppel-like factors and control of energy homeostasis. Endocr. Rev..

[42] Zaragosi LE, Wdziekonski B, Villageois P, Keophiphath M, Maumus M, Tchkonia T, Bourlier V, Mohsen-Kanson T, Ladoux A, Elabd C (2010). Activin a plays a critical role in proliferation and differentiation of human adipose progenitors. Diabetes.

[43] Rossmeislova L, Malisova L, Kracmerova J, Tencerova M, Kovacova Z, Koc M, Siklova-Vitkova M, Viquerie N, Langin D, Stich V (2013). Weight loss improves the adipogenic capacity of human preadipocytes and modulates their secretory profile. Diabetes.

[44] Caso G, McNurlan MA, Mileva I, Zemlyak A, Mynarcik DC, Gelato MC (2013). Peripheral fat loss and decline in adipogenesis in older humans. Metabolism.

[45] Tchoukalova Y, Koutsari C, Jensen M (2007). Committed subcutaneous preadipocytes are reduced in human obesity. Diabetologia.

[46] Clement CC, Santambrogio L (2013). The lymph self-antigen repertoire. Front. Immunol..

[47] Escobedo N, Proulx ST, Karaman S, Dillard ME, Johnson N, Detmar M, Oliver G (2016). Restoration of lymphatic function rescues obesity in Prox1-haploinsufficient mice. JCI Insight.

[48] Engin AB (2017). MicroRNA and adipogenesis. Adv. Exp. Med. Biol..

[49] Thomou T, Mori MA, Dreyfuss JM, Konishi M, Sakaguchi M, Wolfrum C, Rao TN, Winnay JN, Garcia-Martin R, Grinspoon SK (2017). Adipose-derived circulating miRNAs regulate gene expression in other tissues. Nature.

[50] Aldrich MB, Guilliod R, Fife CE, Maus EA, Smith L, Rasmussen JC, Sevick-Muraca EM (2012). Lymphatic abnormalities in the normal contralateral arms of subjects with breast cancer-related lymphedema as assessed by near-infrared fluorescent imaging. Biomed. Opt. Express.

[51] Arner P, Andersson DP, Backdahl J, Dahlman I, Ryden M (2018). Weight Gain and Impaired Glucose Metabolism in Women Are Predicted by Inefficient Subcutaneous Fat Cell Lipolysis. Cell Metab.

[52] Carpenter AE, Jones TR, Lamprecht MR, Clarke C, Kang IH, Friman O, Guertin DA, Chang JH, Lindquist RA, Moffat J (2006). Cell Profiler: image analysis software for identifying and quantifying cell phenotypes. Genome Biol..

[53] Čížková T, Štěpán M, Daďová K, Ondrůjová B, Sontáková L, Krauzová E, Matouš M, Koc M, Gojda J, Kračmerová J (2020). Exercise training reduces inflammation of adipose tissue in the elderly: cross-sectional and randomized interventional trial. J. Clin. Endocrinol. Metab..

[54] Schindelin J, Arganda-Carreras I, Frise E, Kaynig V, Longair M, Pietzsch T, Preibisch S, Rueden C, Saalfeld S, Schmid B (2012). Fiji: an open-source platform for biological-image analysis. Nat. Methods.

[55] Malisova L, Kovacova Z, Koc M, Kracmerova J, Stich V, Rossmeislova L (2013). Ursodeoxycholic acid but not tauroursodeoxycholic acid inhibits proliferation and differentiation of human subcutaneous adipocytes. PLoS ONE.

[56] Brezinova M, Cajka T, Oseeva M, Stepan M, Dadova K, Rossmeislova L, Matous M, Siklova M, Rossmeisl M, Kuda O (2019). Exercise training induces insulin-sensitizing PAHSAs in adipose tissue of elderly women. Biochim. Biophys. Acta Mol. Cell Biol. Lipids.

[57] Chong J, Xia J (2020). Using MetaboAnalyst 4.0 for metabolomics data analysis, interpretation, and integration with other omics data. Methods Mol. Biol..

