# Supplementary material for: Lymphedema alters lipolytic, lipogenic, immune and angiogenic properties of adipose tissue: a hypothesis-generating study in breast cancer survivors
Source: Sci Rep. 2021 Apr 14;11:8171. doi: 10.1038/s41598-021-87494-3 (PMC8046998; doi:10.1038/s41598-021-87494-3)
Supplement: Supplementary file 1 — Supplementary Information 1. [file 41598_2021_87494_MOESM1_ESM.docx]

Lymphedema alters lipolytic, lipogenic, immune and angiogenic properties of adipose tissue: a hypothesis-generating study in breast cancer survivors.

Michal Koc, Martin Wald, Zuzana Varaliová, Barbora Ondrůjová, Terezie Čížková, Milan Brychta, Jana Kračmerová, Lenka Beranová, Jan Pala, Veronika Šrámková, Michaela Šiklová, Jan Gojda and Lenka Rossmeislová

Supplementary Table 2- List of genes analysed in AT and adipose cells

| **Gene symbol** | **Assay ID** | **Gene name** | **Preadipocytes and adipocytes in vitro** | **AT** |
| --- | --- | --- | --- | --- |
| ACACA | Hs01046047_m1 | acetyl-CoA carboxylase alpha |  | ● too high CT, excluded |
| ACLY | Hs00982738_m1 | ATP citrate lyase | ● |  |
| ADA | Hs01110945_m1 | adenosine deaminase |  | ● |
| ADIPOQ | Hs00605917_m1 | Adiponectin | ● | ● |
| ADRB2 | Hs00240532_s1 | Adrenergic, beta-2-, receptor, surface |  | ● |
| ANGPTL3 | Hs00205581_m1 | angiopoietin like 3 |  | ● too high CT, excluded |
| ANGPTL4 | Hs01101127_m1 | Angiopoietin-like 4 | ● | ● |
| BAMBI | Hs03044164_m1 | BMP and activin membrane bound inhibitor | ● | ● |
| CCL5=RANTES | Hs00174575_m1 | chemokine (C-C motif) ligand 5 |  | ● |
| CD14 | Hs00169122_g1 | CD14 molecule |  | ● |
| CD36 | Hs00354519_m1 | CD36 molecule | ● | ● |
| CD3e | Hs01062241_m1 | CD3e molecule, epsilon |  | ● |
| CD4 | Hs01058407_m1 | CD4 molecule |  | ● |
| CD8a | Hs00233520_m1 | CD8a molecule |  | ● |
| CDKN1A (P21) | Hs00355782_m1 | cyclin dependent kinase inhibitor 1A |  | ● |
| CHREBP | Hs00975714_m1 | MLX interacting protein-like | ● | ● |
| COL1A1 | Hs00164004_m1 | collagen, type I, alpha 1 |  | ● High SD, excluded |
| COL6A3 | Hs00365098_m1 | Collagen, type VI, alpha 3 | ● | ● |
| DCN | Hs00754870_s1 | Decorin | ● | ● |
| DDIT3 | Hs01090850_m1 | DNA-damage-inducible transcript 3 |  | ● |
| DGAT2 | Hs01045913_m1 | Diacylglycerol O-acyltransferase 2 | ● | ● |
| DUSP1 | Hs00610256_g1 | Dual specificity phosphatase 1 |  | ● |
| ECM1 | Hs00189435_m1 | Extracellular matrix protein 1 | ● | ● |
| ELOVL6 | Hs00225412_m1 | ELOVL fatty acid elongase 6 |  | ● |
| FABP4 | Hs01086177_m1 | Fatty acid binding protein 4 | ● |  |
| FASN | Hs01005622_m1 | Fatty acid synthase | ● | ● |
| FGF1 | Hs01092738_m1 | Fibroblast growth factor 1 | ● |  |
| FIBRO | Hs00365052_m1 | Fibronectin 1 | ● | ● |
| GDF15 | Hs00171132_m1 | Growth differentiation factor 15 | ● | ● |
| GLUT4 (SLC2A4) | Hs00168966_m1 | Glucose transporter 4 |  | ● |
| GOT2 | Hs00905827_g1 | Glutamic-oxaloacetic transaminase 2 |  | ● |
| **GUSB** | Hs00939627_m1 | glucuronidase, beta |  | ● |
| **HAND2** | Hs00232769_m1 | heart and neural crest derivatives expressed 2 |  | ● |
| HIF1A | Hs00153153_m1 | Hypoxia inducible factor 1, alpha subunit |  | ● |
| HSD11B1 | Hs01547870_m1 | Hydroxysteroid (11-beta) dehydrogenase 1 | ● | ● |
| HSL (LIPE) | Hs00943404_m1 | Lipase, hormone-sensitive |  | ● |
| HYOU1 | Hs00197328_m1 | hypoxia up-regulated 1 |  | ● |
| ICAM1 | Hs00164932_m1 | intercellular adhesion molecule 1 |  | ● |
| IL1b | Hs01555410_m1 | Interleukin 1, beta |  | ● |
| IL2RA (CD25) | Hs00907779_m1 | Interleukin 2 receptor, alpha |  | ● |
| IL6 | Hs00985639_m1 | Interleukin 6 (interferon, beta 2) |  | ● |
| IL8 | Hs00174103_m1 | Interleukin 8 |  | ● |
| INHBA | Hs01081598_m1 | Inhibin beta A subunit | ● | ● |
| IRF1 | Hs00971960_m1 | Interferon regulatory factor 1 | ● | ● |
| IRF5 | Hs00158114_m1 | interferon regulatory factor 5 |  | ● |
| ITGB1 | Hs00559595_m1 | Integrin, beta 1 | ● | ● |
| ITGB2 | Hs00164957_m1 | integrin, beta 2 (complement component 3 receptor 3 and 4 subunit) | ● | ● |
| KLF4 | Hs00358836_m1 | Kruppel-like factor 4 | ● | ● |
| KLF6 | Hs00810569_m1 | Kruppel-like factor 6 | ● |  |
| KLF9 | Hs00230918_m1 | Kruppel-like factor 9 |  | ● |
| LDHA | Hs01378790_g1 | lactate dehydrogenase A |  | ● |
| LEP | Hs00174877_m1 | Leptin | ● | ● |
| LOX | Hs00942480_m1 | Lysyl oxidase | ● | ● |
| LUM | Hs00929860_m1 | Lumican | ● | ● |
| LYVE-1 | Hs00272659_m1 | Lymphatic Vessel Endothelial Hyaluronan Receptor 1 |  | ● High SD, excluded |
| MCP1 | Hs00234140_m1 | Chemokine (C-C motif) ligand 2 |  | ● |
| MMP2 | Hs01548727_m1 | Matrix metallopeptidase 2 | ● | ● |
| MMP9 | Hs00234579_m1 | Matrix metallopeptidase 9 |  |  |
| MMP19 | Hs00275699_m1 | Matrix metallopeptidase 19 |  | ● |
| MRC1=CD206 | Hs00267207_m1 | Mannose receptore C type 1 |  | ● |
| NDN | Hs00267349_s1 | Necdin, MAGE family member | ● | ● |
| NOX4 | Hs01379108_m1 | NADPH oxidase 4 |  |  |
| NRF1 | Hs00192316_m1 | Nuclear respiratory factor 1 | ● | ● |
| p16 (CDKN2A) | Hs00923894_m1 | Cyclin-dependent kinase inhibitor 2A |  | ● |
| PDK4 | Hs01037712_m1 | Pyruvate dehydrogenase kinase 4 |  | ● |
| PDPN | Hs00366766_m1 | Podoplanin |  | ● |
| PECAM | Hs01065282_m1 | Platelet/endothelial cell adhesion molecule 1 | ● | ● |
| PGC1 | Hs01016719_m1 | Peroxisome proliferator-activated receptor gamma, coactivator 1 alpha | ● | ● |
| PLIN | Hs00160173_m1 | Perilipin | ● | ● |
| PLIN2 | Hs00605340_m1 | Perilipin 2 |  | ● |
| PLIN3 | Hs00998416_m1 | Perilipin 3 |  | ● |
| PNPLA2 (ATGL) | Hs00982040_g1 | Patatin-like phospholipase domain containing 2 | ● | ● |
| PPARG | Hs01115513_m1 | Peroxisome proliferator-activated receptor gamma | ● | ● |
| PPARGC1A (PGC1) | Hs01016719_m1 | Peroxisome proliferator-activated receptor gamma, coactivator 1 alpha | ● |  |
| PPIA | Hs04194521_s1 | Peptidylprolyl isomerase A |  | ● |
| PROX1 | Hs00896294_m1 | prospero homeobox 1 |  | ● |
| RPS13 | Hs01011487_g1 | Ribosomal protein S13 | ● | ● |
| RUNX2 | Hs00231692_m1 | Runt-related transcription factor 2 | ● | ● |
| SCD | Hs01682761_m1 | Stearoyl-CoA desaturase | ● | ● |
| SLC27A1 | Hs01587911_m1 | Solute carrier family 27 member 1 | ● | ● |
| SLC27A2 | Hs00186324_m1 | solute carrier family 27 member 2 |  | ● High SD, excluded |
| SPARC | Hs00234160_m1 | Secreted protein, acidic, cysteine-rich | ● | ● |
| SREBF1 | Hs01088691_m1 | Sterol regulatory element binding transcription factor 1 |  | ● |
| TBP | Hs00427620_m1 | TATA box binding protein | ● | ● |
| TFAM | Hs01082775_m1 | transcription factor A, mitochondrial | ● |  |
| TGFb1 | Hs00998133_m1 | Transforming growth factor, beta 1 | ● | ● |
| TGFb3 | Hs01086000_m1 | transforming growth factor, beta 3 | ● |  |
| TIMP1 | Hs00171558_m1 | Tissue inhibitor of metalloproteinase 1 |  | ● |
| TIMP2 | Hs00234278_m1 | TIMP metallopeptidase inhibitor 2 |  | ● |
| TIMP3 | Hs00165949_m1 | TIMP metallopeptidase inhibitor 3 |  | ● |
| TLR4 | Hs01060206_m1 | Toll-like receptor 4 |  | ● |
| TNC | Hs01115665_m1 | Tenascin C | ● | ● |
| VEGFA | Hs00900055_m1 | Vascular endothelial growth factor A | ● | ● |
| VEGFC | Hs01099203_m1 | Vascular endothelial growth factor C | ● | ● |
| VEGFD | Hs01128657_m1 | Vascular endothelial growth factor D | ● | ● |
| VEGFR2 | Hs00911700_m1 | kinase insert domain receptor |  | ● |
| VEGFR4 | Hs00176607_m1 | fms-related tyrosine kinase 4 |  | ● |
| WISP2 | Hs00180242_m1 | WNT1 inducible signaling pathway protein 2 | ● | ● |
| ZNF423 | Hs00323880_m1 | Zinc finger protein 423 | ● | ● |
