# Supplementary material for: Lymphedema alters lipolytic, lipogenic, immune and angiogenic properties of adipose tissue: a hypothesis-generating study in breast cancer survivors
Source: Sci Rep. 2021 Apr 14;11:8171. doi: 10.1038/s41598-021-87494-3 (PMC8046998; doi:10.1038/s41598-021-87494-3)
Supplement: Supplementary file 2 — Supplementary Information 2. [file 41598_2021_87494_MOESM2_ESM.docx]

Lymphedema alters lipolytic, lipogenic, immune and angiogenic properties of adipose tissue: a hypothesis-generating study in breast cancer survivors.

Michal Koc, Martin Wald, Zuzana Varaliová, Barbora Ondrůjová, Terezie Čížková, Milan Brychta, Jana Kračmerová, Lenka Beranová, Jan Pala, Veronika Šrámková, Michaela Šiklová, Jan Gojda and Lenka Rossmeislová

Supplementary Table 2 Correlations among mRNA levels of PDPN, HAND2, NDN and PECAM1 and mRNA levels of other genes, lipolysis measures and results of ex vivo angiogenic assay. Spearman coefficient was calculated. Only those correlations with p value below 0.05 are shown.

|  | mRNA PDPN | | mRNA HAND2 | | mRNA NDN | | mRNA PECAM1 | |
| --- | --- | --- | --- | --- | --- | --- | --- | --- |
|  | R | p value | R | p value | R | p value | R | p value |
| ADA |  |  |  |  | 0.530 | 0.00152 | -0.345 | 0.04932 |
| ADRB2 | -0.410 | 0.01788 | -0.498 | 0.00319 | 0.482 | 0.00454 |  |  |
| ANGPTL4 | 0.427 | 0.01309 |  |  |  |  |  |  |
| CCL5(RANTES) | 0.665 | 0.00002 | 0.627 | 0.00009 | -0.616 | 0.00014 | 0.547 | 0.00098 |
| CD36 |  |  |  |  |  |  | 0.486 | 0.00417 |
| CD3e | 0.637 | 0.00007 | 0.484 | 0.00435 | -0.565 | 0.00062 | 0.602 | 0.00021 |
| CD4 | 0.418 | 0.01555 |  |  |  |  | 0.530 | 0.00150 |
| CD8a | 0.634 | 0.00008 | 0.602 | 0.00021 | -0.603 | 0.00020 | 0.561 | 0.00068 |
| CHREBP(MLXIPL) | -0.402 | 0.02037 | -0.479 | 0.00484 | 0.604 | 0.00020 | -0.470 | 0.00579 |
| COL6A3 |  |  |  |  |  |  | 0.380 | 0.02931 |
| DUSP1 | -0.358 | 0.04082 | -0.606 | 0.00018 | 0.542 | 0.00113 |  |  |
| ECM1 | 0.438 | 0.01083 | 0.531 | 0.00147 | -0.366 | 0.03621 |  |  |
| ELOVL6 | -0.509 | 0.00247 | -0.582 | 0.00038 | 0.558 | 0.00074 |  |  |
| FIBRO | 0.636 | 0.00007 | 0.566 | 0.00060 | -0.483 | 0.00445 | 0.584 | 0.00036 |
| FLT4 (VEGFR3) |  |  |  |  |  |  | 0.408 | 0.01829 |
| GDF15 | 0.349 | 0.04657 | 0.413 | 0.01678 |  |  |  |  |
| GLUT4 |  |  | 0.358 | 0.04082 |  |  | 0.400 | 0.02118 |
| GOT2 | 0.601 | 0.00022 | 0.505 | 0.00270 | -0.456 | 0.00767 | 0.652 | 0.00004 |
| GUSB |  |  |  |  | 0.384 | 0.02736 |  |  |
| HAND2 | 0.644 | 0.00005 |  |  | -0.828 | 0.00000 | 0.420 | 0.01501 |
| HIF1A | 0.519 | 0.00195 |  |  |  |  | 0.561 | 0.00068 |
| HSD11B1 |  |  | 0.486 | 0.00417 | -0.379 | 0.02977 |  |  |
| HSL (LIPE) | 0.353 | 0.04415 | 0.439 | 0.01069 |  |  | 0.512 | 0.00232 |
| HYOU1 | 0.516 | 0.00213 |  |  |  |  | 0.533 | 0.00139 |
| ICAM1 | 0.804 | 0.00000 | 0.608 | 0.00017 | -0.697 | 0.00001 | 0.561 | 0.00068 |
| IL1B | 0.503 | 0.00285 |  |  |  |  | 0.397 | 0.02214 |
| INHBA |  |  | 0.352 | 0.04458 |  |  | 0.555 | 0.00080 |
| IRF1 | 0.757 | 0.00000 | 0.699 | 0.00001 | -0.681 | 0.00001 | 0.552 | 0.00086 |
| ITGB1 |  |  | 0.712 | 0.00000 | -0.453 | 0.00813 | 0.344 | 0.04979 |
| ITGB2 | 0.369 | 0.03477 | 0.388 | 0.02566 |  |  |  |  |
| KDR (VEGFR2) (FLK1) |  |  |  |  |  |  | 0.368 | 0.03512 |
| KLF9 |  | 0.44924 |  | 0.94554 |  | 0.34005 | 0.433 | 0.01180 |
| LDHA | 0.483 | 0.00445 | 0.421 | 0.01475 | -0.369 | 0.03477 | 0.398 | 0.02190 |
| LEP |  |  | 0.538 | 0.00124 | -0.392 | 0.02417 | 0.368 | 0.03530 |
| LOX |  |  | 0.463 | 0.00672 |  |  |  |  |
| MMP19 | 0.382 | 0.02839 | 0.353 | 0.04373 |  |  | 0.390 | 0.02484 |
| MRC1=CD206 |  |  |  |  | 0.378 | 0.03024 |  |  |
| NDN | -0.764 | 0.00000 | -0.828 | 0.00000 |  |  | -0.570 | 0.00053 |
| NRF1 | 0.378 | 0.03024 |  |  |  |  | 0.577 | 0.00044 |
| PDK4 |  |  | -0.419 | 0.01510 |  |  |  |  |
| PDPN |  |  | 0.644 | 0.00005 | -0.764 | 0.00000 | 0.562 | 0.00066 |
| PECAM1 | 0.562 | 0.00066 | 0.420 | 0.01501 | -0.570 | 0.00053 |  |  |
| PLIN | 0.601 | 0.00022 | 0.628 | 0.00009 | -0.621 | 0.00012 | 0.576 | 0.00045 |
| PLIN2 | -0.690 | 0.00001 | -0.602 | 0.00021 | 0.713 | 0.00000 | -0.602 | 0.00021 |
| PLIN3 | 0.621 | 0.00012 | 0.702 | 0.00001 | -0.579 | 0.00042 | 0.414 | 0.01658 |
| PNPLA2 (ATGL) | -0.673 | 0.00002 | -0.682 | 0.00001 | 0.841 | 0.00000 | -0.661 | 0.00003 |
| PPARG | -0.408 | 0.01829 |  |  | 0.365 | 0.03676 |  |  |
| PPARGC1A (PGC1) | -0.428 | 0.01296 | -0.469 | 0.00593 | 0.468 | 0.00597 |  |  |
| PROX1 |  |  |  |  |  |  | 0.405 | 0.01925 |
| SCD |  |  |  |  |  |  | 0.350 | 0.04612 |
| SLC27A1 |  |  | -0.355 | 0.04288 | 0.415 | 0.01620 |  |  |
| SLC27A2 | -0.577 | 0.00044 | -0.574 | 0.00048 | 0.555 | 0.00081 |  |  |
| SPARC |  |  | 0.498 | 0.00316 | -0.390 | 0.02497 | 0.436 | 0.01110 |
| SREBF1 | -0.438 | 0.01083 | -0.507 | 0.00260 | 0.424 | 0.01382 |  |  |
| TGFb1 | 0.633 | 0.00008 | 0.602 | 0.00021 | -0.520 | 0.00194 | 0.697 | 0.00001 |
| TIMP1 | 0.701 | 0.00001 | 0.637 | 0.00007 | -0.571 | 0.00053 | 0.584 | 0.00036 |
| TIMP2 | 0.709 | 0.00000 | 0.621 | 0.00012 | -0.636 | 0.00007 | 0.630 | 0.00009 |
| TIMP3 |  |  |  |  |  |  | 0.364 | 0.03713 |
| TLR4 | -0.449 | 0.00873 | -0.561 | 0.00068 | 0.659 | 0.00003 |  |  |
| TNC | 0.826 | 0.00000 | 0.692 | 0.00001 | -0.777 | 0.00000 | 0.551 | 0.00089 |
| VEGFA |  |  | -0.473 | 0.00541 | 0.480 | 0.00474 |  |  |
| WISP2 | 0.805 | 0.00000 | 0.707 | 0.00000 | -0.634 | 0.00007 | 0.431 | 0.01224 |
| ZNF423 | 0.507 | 0.00260 |  |  |  | 0.06422 | 0.683 | 0.00001 |
| angiosprouts | 0.652 | 0.00023 |  |  | -0.593 | 0.00112 |  |  |
| GLY basal | 0.846 | 0.00000 | 0.815 | 0.00000 | -0.848 | 0.00000 | 0.591 | 0.00376 |
| FFA:GLY basal | 0.771 | 0.00004 | 0.668 | 0.00095 | -0.747 | 0.00010 | 0.594 | 0.00456 |
| FFA basal | 0.655 | 0.00006 | 0.664 | 0.00005 | -0.517 | 0.00289 |  |  |
