# Supplementary material for: Lymphedema alters lipolytic, lipogenic, immune and angiogenic properties of adipose tissue: a hypothesis-generating study in breast cancer survivors
Source: Sci Rep. 2021 Apr 14;11:8171. doi: 10.1038/s41598-021-87494-3 (PMC8046998; doi:10.1038/s41598-021-87494-3)
Supplement: Supplementary file 3 — Supplementary Information 3. [file 41598_2021_87494_MOESM3_ESM.docx]

Lymphedema alters lipolytic, lipogenic, immune and angiogenic properties of adipose tissue: a hypothesis-generating study in breast cancer survivors.

Michal Koc, Martin Wald, Zuzana Varaliová, Barbora Ondrůjová, Terezie Čížková, Milan Brychta, Jana Kračmerová, Lenka Beranová, Jan Pala, Veronika Šrámková, Michaela Šiklová, Jan Gojda and Lenka Rossmeislová

| Subjects with secondary lymphedema (n=11) Cancer survivors without lymphedema (n=11) | | | |
| --- | --- | --- | --- |
| Radiotherapy |  | n = 11 | n = 9 |
| Chemotherapy |  | n = 10 | n = 6 |
| Hormonal therapy |  | n = 4 | n = 6 |
| Hormonal therapy at the time of sample acquisition |  | n = 1 | n = 2 |

Supplementary Table 3. Breast cancer therapy following the primary surgery (partial or total mastectomy). Therapy was combined in majority of cases. Number of women undergoing specified treatment are shown.
