# Supplementary figures and images for: Lymphedema alters lipolytic, lipogenic, immune and angiogenic properties of adipose tissue: a hypothesis-generating study in breast cancer survivors
Source: Sci Rep. 2021 Apr 14;11:8171. doi: 10.1038/s41598-021-87494-3 (PMC8046998; doi:10.1038/s41598-021-87494-3)

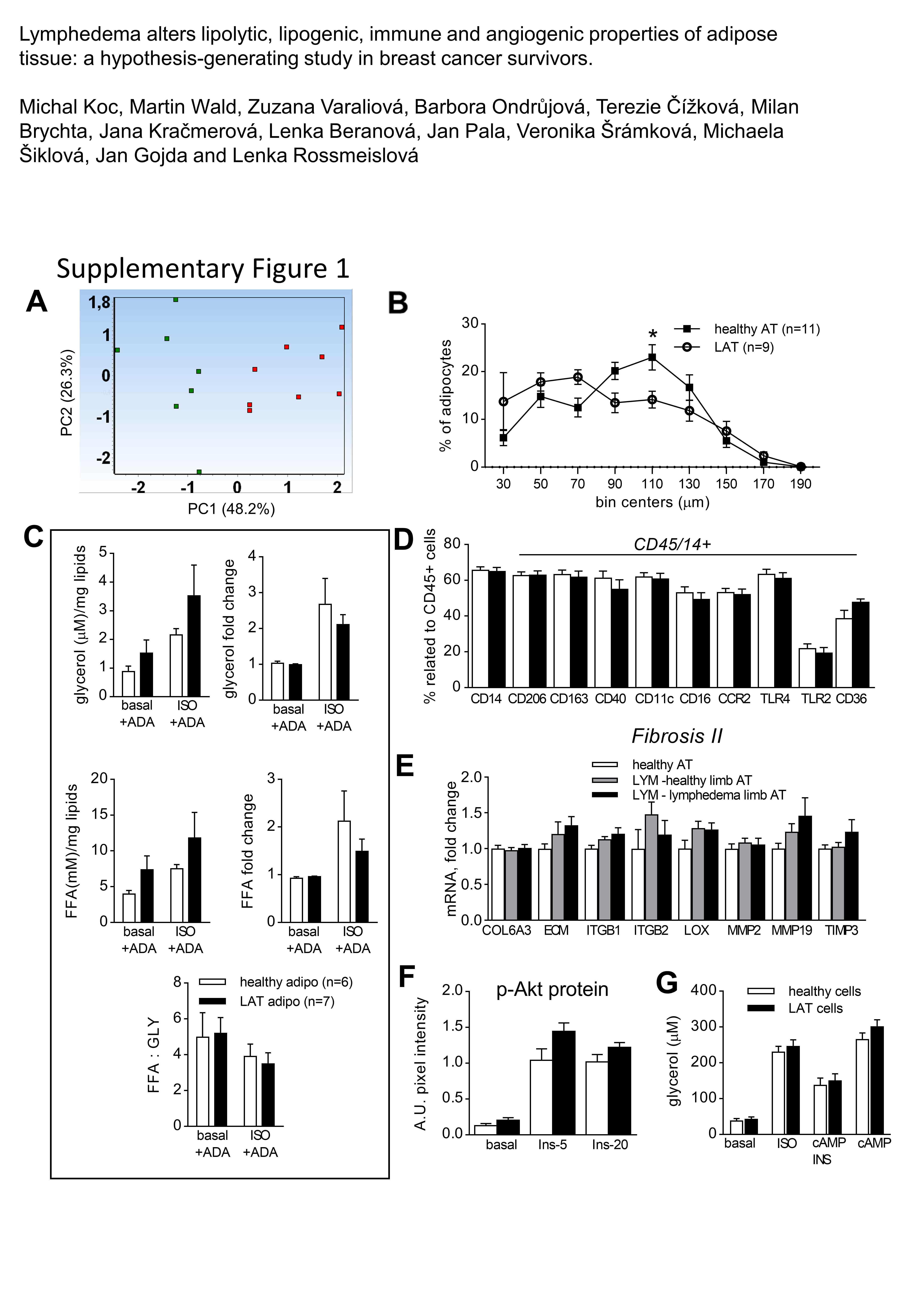

Supplement: Supplementary file 6 — Supplementary Information 6. [file 41598_2021_87494_MOESM6_ESM.tif]

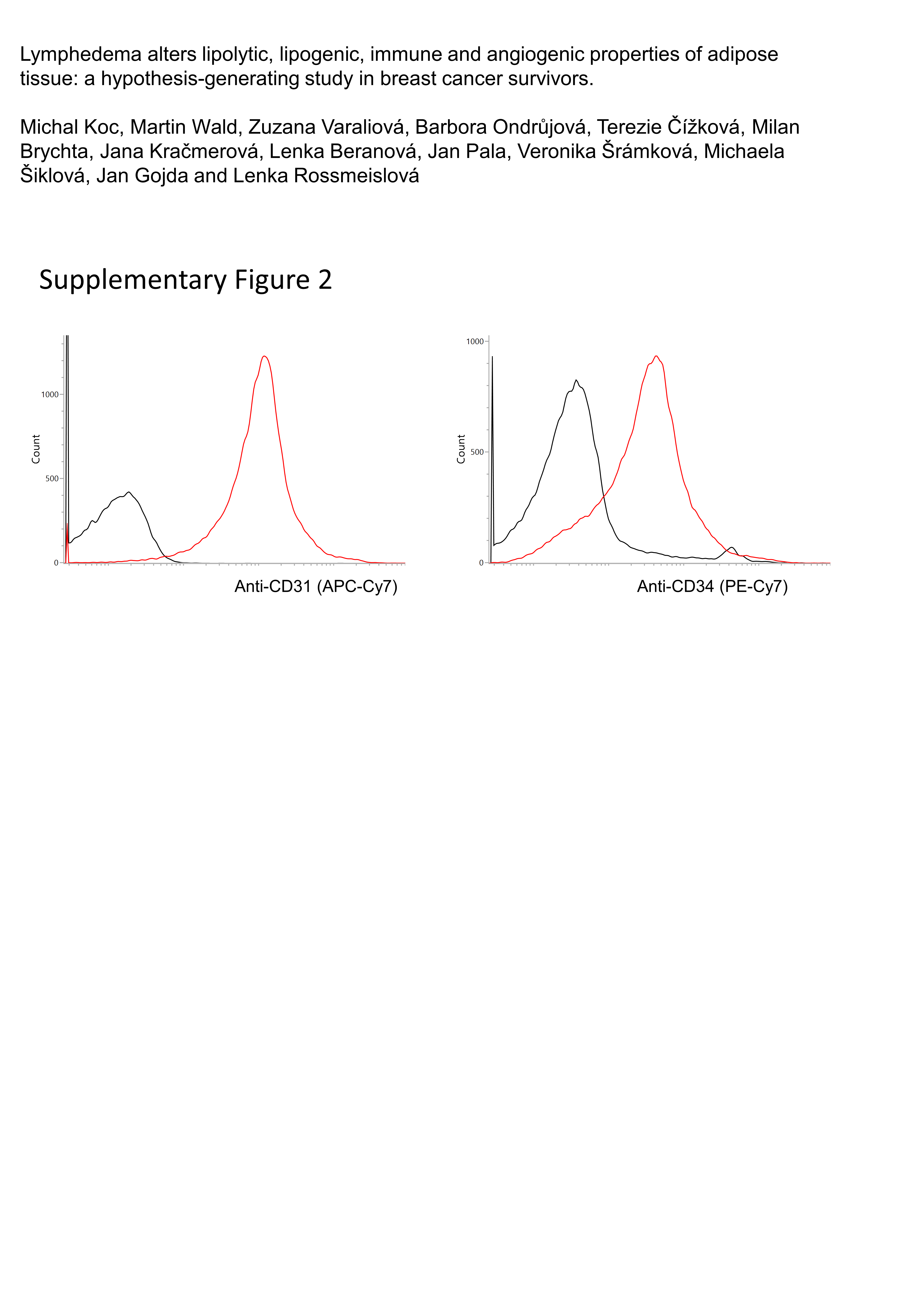

Supplement: Supplementary file 7 — Supplementary Figure 1: Effects of lymphedema on systemic variables and AT qualities. (A) The 2D score plot of the PCA of miRNA expression that characterizes the trends exhibited by the expression profiles of LYM (red) and non-lymphedema subjects (combined heathy and NOLYM, green). Each dot represents a subject. The degree of variability is expressed as a percentage. (B) Distribution of adipocyte size. Histogram of frequencies of cells within 20 µm bins (Two-way ANOVA, Sidak post-hoc analysis, *p <0.05). (C) Ex vivo lipolysis in isolated adipocytes. Concentrations of glycerol and FFA normalized to mg of lipids as well as fold change over the basal conditions are shown. ISO-1µM isoproterenol, ADA, adenosine deaminase (Two-way ANOVA of LN transformed data, Sidak post-hoc analysis). (D) Relative content of macrophage populations in AT, expressed as percentage of CD45+ positive cells (Mann Whitney test). (E) mRNA levels in whole AT expressed as fold change over the mean expression of healthy group (Kruskal-Wallis test of 2ΔCt values, Dunn’s correction, comparison of 3 groups; Wilcoxon test of paired healthy and diseased limb of LYM subjects).(F) Phosphorylation of Akt in lysates from adipocytes exposed to insulin for 5 or 20 minutes. Signal for p-Akt antibody was normalized to total Akt signal. Ins, 100 nM insulin. (G) Analysis of in vitro lipolysis in adipocytes. Cells were exposed to basal conditions, isoproterenol, 8-Br-cAMP or insulin for 3 hours. ISO-1µM isoproterenol, cAMP, 1 mM 8-Br-cAMP, INS, 1nM insulin (Two-way ANOVA of LN transformed data, Sidak post-hoc analysis). [file 41598_2021_87494_MOESM7_ESM.tif]

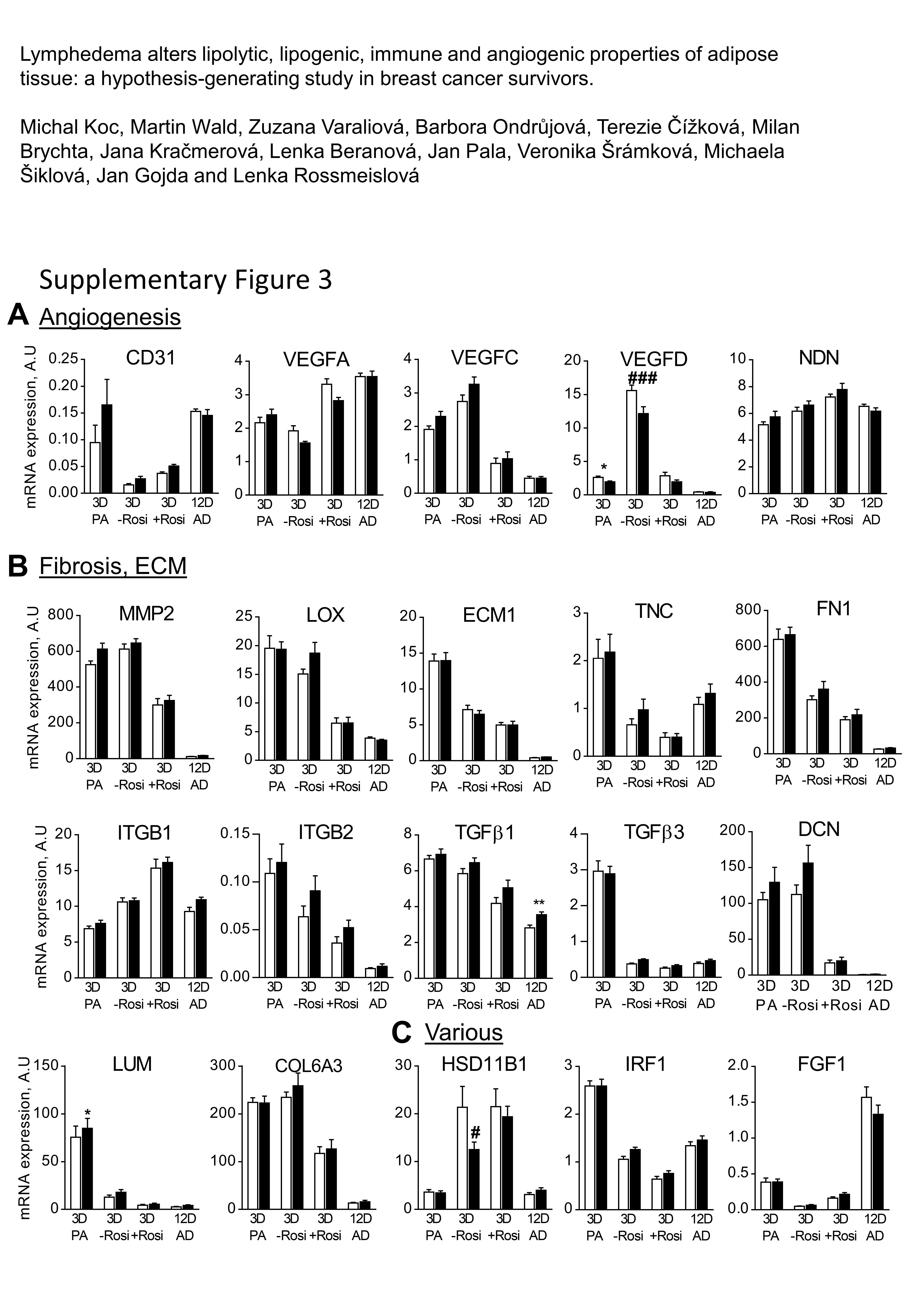

Supplement: Supplementary file 8 — Supplementary Figure 2: Flow cytometry analysis of cells originating from endothelial sprouts. Cells were extracted from Matrigel upon the termination of the angiogenic assay and subcultivated in EGM2 medium. Then they were detached from the plastic culture ware by trypsinization, collected, stained for CD31 and CD34 and analysed by flow cytometry. The representative histograms showing the negative controls (unstained cells, black line) and stained cells (red line) are shown. [file 41598_2021_87494_MOESM8_ESM.tif]

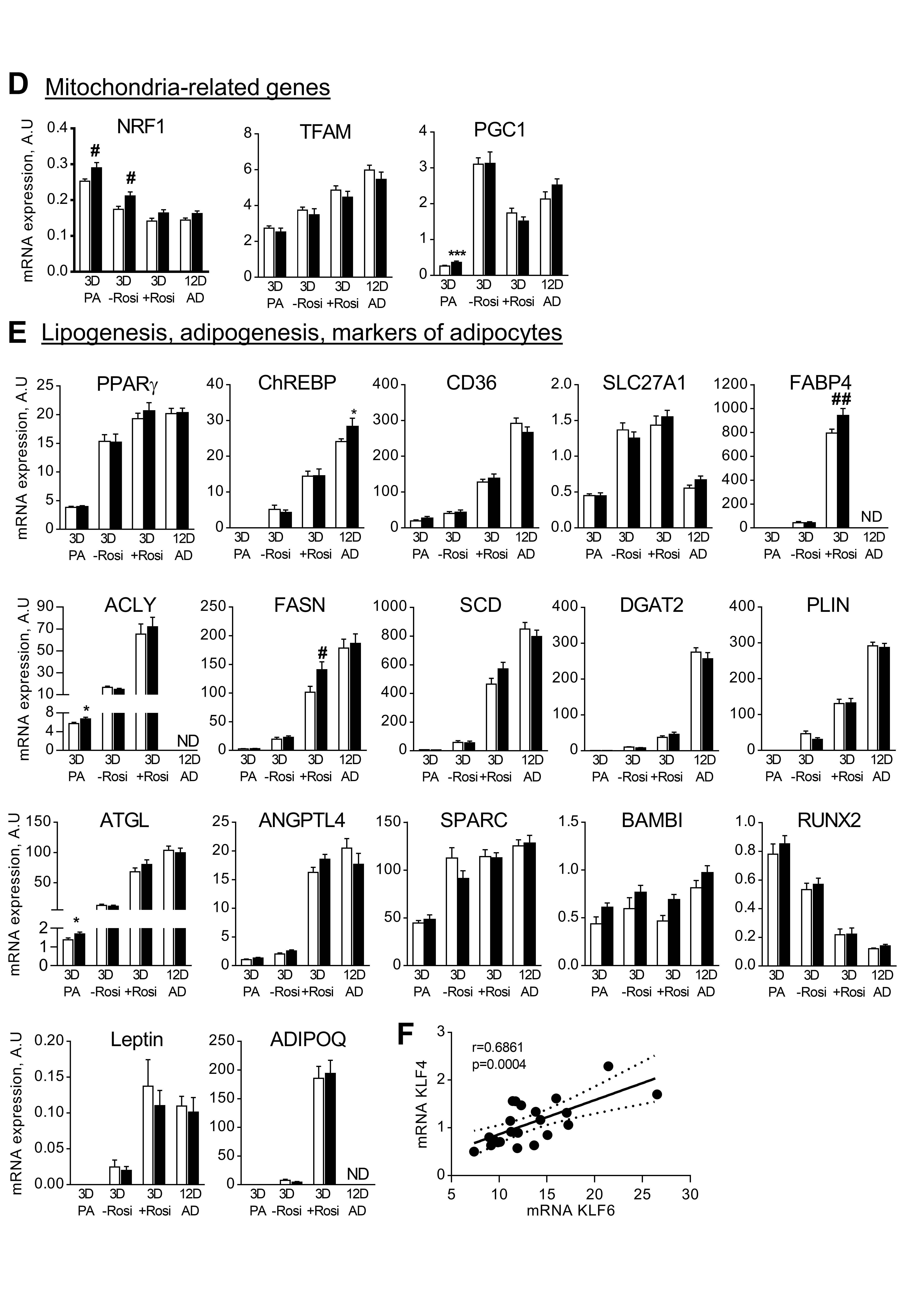

Supplement: Supplementary file 9 — Supplementary Figure 3: mRNA expression of selected genes in preadipocytes, early and mature adipocytes under in vitro conditions. Preadipocytes (PA, 3D) were harvested after 3 days in basal medium (serum free), early adipocytes after exposition to adipogenic medium (serum free) lacking or containing Rosiglitazone for 3 days (3D plus or minus Rosi) and mature adipocytes after completed adipogenic protocol (12 days, 12D AD). (A) Angiogenic markers; (B) Markers of fibrosis and extracellular matrix (ECM); (C) Various; (D) Mitochondria-related genes; (E) Markers of lipogenesis and adipogenesis. Expression levels were calculated as 2ΔCt (Two-way ANOVA, Sidak post-hoc analysis, #p<0.05, ###p<0.001; Mann Whitney test of expression in preadipocytes and mature adipocytes analysed individually, *p <0.05). F. Correlation between mRNA levels of KLF4 and KLF6 in preadipocytes (Spearman correlation coefficient). [file 41598_2021_87494_MOESM9_ESM.tif]

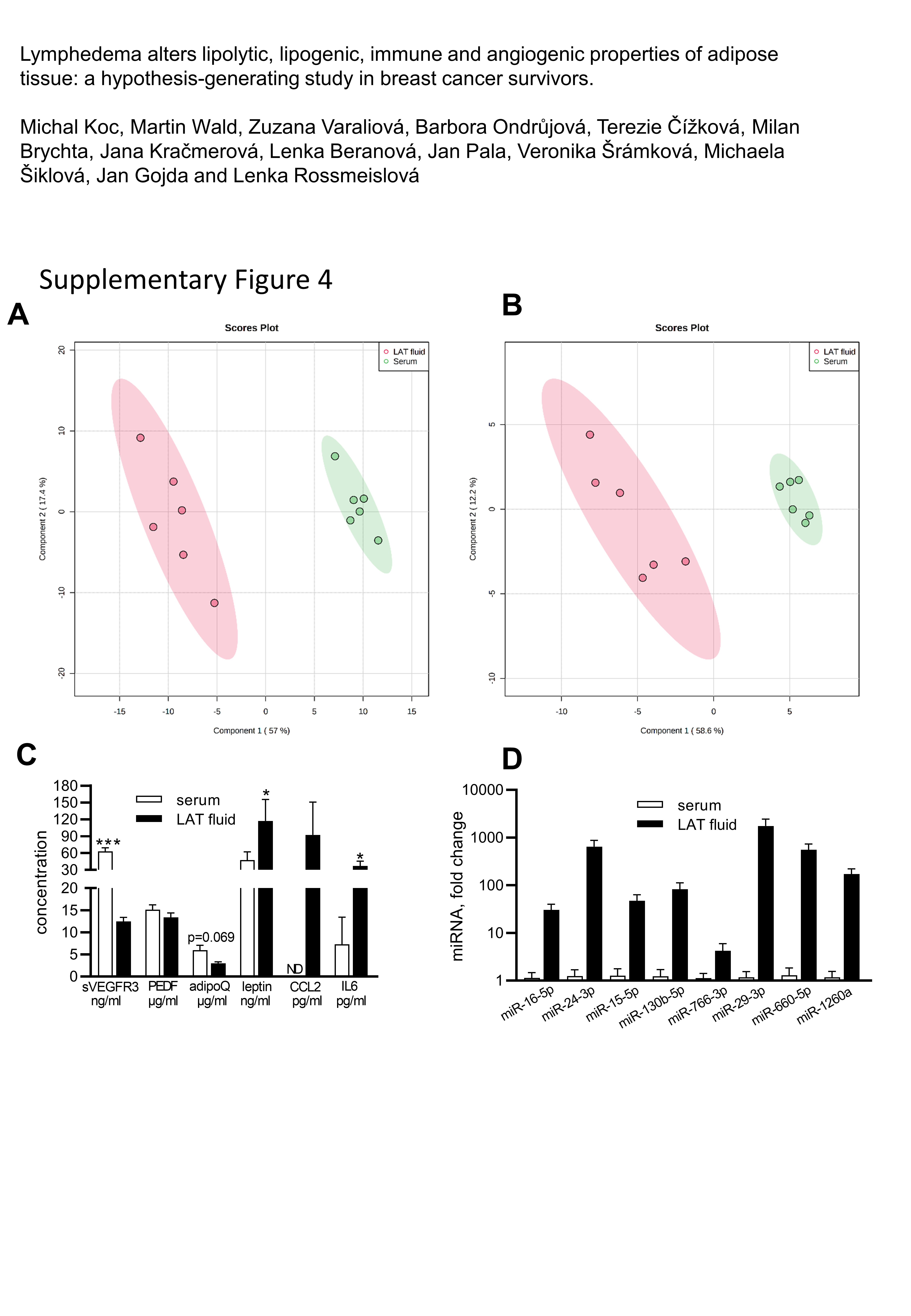

Supplement: Supplementary file 10 — Supplementary Figure 4: Comparison of composition of LAT fluid vs. paired serum. PLS-DA score scatter plot of the identified lipids (A) and polar metabolites (B) reveals a clear separation between serum and LAT fluid (n=6); (C) Cytokine levels, measured by ELISA (n=6, paired t test, *p <0.05, *** p<0.001, ND-not detectable); (D) miRNA levels measured by qPCR (n=4). miR-16-5p, miR-24-3p and miR-15-5p were originally intended as endogenous normalizers, since they have stable expression in human serum, but they could not be used for normalization of miRNAs of interest in both LAT fluid and serum due to their substantially higher expression in LAT. [file 41598_2021_87494_MOESM10_ESM.tif]
